# Viral Glycoprotein Complex Formation, Essential Function and Immunogenicity in the Guinea Pig Model for Cytomegalovirus

**DOI:** 10.1371/journal.pone.0135567

**Published:** 2015-08-12

**Authors:** Stewart Coleman, Julia Hornig, Sarah Maddux, K. Yeon Choi, Alistair McGregor

**Affiliations:** Department of Microbial Pathogenesis & Immunology, Texas A&M University, Health Science Center, College of Medicine, College Station, TX, United States of America; Novartis Vaccines, UNITED STATES

## Abstract

Development of a cytomegalovirus (CMV) vaccine is a major public health priority due to the risk of congenital infection. A key component of a vaccine is thought to be an effective neutralizing antibody response against the viral glycoproteins necessary for cell entry. Species specificity of human CMV (HCMV) precludes direct studies in an animal model. The guinea pig is the only small animal model for congenital cytomegalovirus infection. Analysis of the guinea pig CMV (GPCMV) genome indicates that it potentially encodes homologs to the HCMV glycoproteins (including gB, gH, gL, gM, gN and gO) that form various cell entry complexes on the outside of the virus: gCI (gB); gCII (gH/gL/gO); gCIII (gM/gN). The gB homolog (GP55) has been investigated as a candidate subunit vaccine but little is known about the other homolog proteins. GPCMV glycoproteins were investigated by transient expression studies which indicated that homolog glycoproteins to gN and gM, or gH, gL and gO were able to co-localize in cells and generate respective homolog complexes which could be verified by immunoprecipitation assays. ELISA studies demonstrated that the individual complexes were highly immunogenic in guinea pigs. The gO (GP74) homolog protein has 13 conserved N-glycosylation sites found in HCMV gO. In transient expression studies, only the glycosylated protein is detected but in virus infected cells both N-glycosylated and non-glycosylated gO protein were detected. In protein interaction studies, a mutant gO that lacked N-glycosylation sites had no impact on the ability of the protein to interact with gH/gL which indicated a potential alternative function associated with these sites. Knockout GPCMV BAC mutagenesis of the respective glycoprotein genes (*GP55* for gB, *GP75* for gH, *GP115* for gL, *GP100* for gM, *GP73* for gN and *GP74* for gO) in separate reactions was lethal for virus regeneration on fibroblast cells which demonstrated the essential nature of the GPCMV glycoproteins. The gene knockout results were similar to HCMV, except in the case of the gO homolog, which was non-essential in epithelial tropic virus but essential in lab adapted GPCMV. Overall, the findings demonstrate the similarity between HCMV and GPCMV glycoproteins and strengthen the relevance of this model for development of CMV intervention strategies.

## Introduction

Congenital human cytomegalovirus (HCMV) infection occurs in approximately 1% of live births in the US and can lead to symptomatic disease including mental retardation and hearing loss [1, 2]. In congenital HCMV infection, the greatest risk is to mothers who acquire a primary infection during pregnancy [3], with an overall fetal transmission rate of 37.1% to 64.1% [4]. It is realistic to expect that a vaccine against HCMV will offer some form of protection against congenital infection since vertical transmission is relatively low in mothers convalescent for HCMV. Consequently, with an estimated level of transmission to sero-negative pregnant women of 27,000 per year in the US [5] the impact of a vaccine could be substantial in reducing the risk for congenital CMV infection. Any proposed intervention therapy for the prevention or treatment of HCMV infection should ideally be evaluated in an animal model. Unfortunately, due to the extreme species specificity of HCMV, studies in animal models are untenable.

Animal model pathogenicity, vaccine and antiviral studies of CMV are carried out with animal-specific CMVs such as guinea pig (GPCMV), mouse (MCMV), rat (RCMV) and rhesus macaques (RhCMV). The genomes of all of these animal CMVs have been sequenced [6–10]. The guinea pig is unique insofar as it is the only small animal model to allow the study of congenital CMV infection. Presumably, this is based on the similarity of placenta structure between human and guinea pig placentas which both are hemochorial containing a homogenous layer of trophoblast cells separating maternal and fetal circulation [11–13]. Importantly, GPCMV congenital infection causes disease in the fetus and in newborn guinea pig pups which are similar to those found in humans, including sensorineural hearing loss [14–16]. Consequently, the guinea pig model is best suited for testing of vaccines or other intervention strategies aimed at preventing congenital CMV infection [17–19].

A drawback in GPCMV and the guinea pig model has been a lack of development at the molecular level. This has largely been overcome by the recent sequencing of the viral genome and the development of infectious BAC clones of the GPCMV genome [9, 10, 20, 21]. Additionally, the guinea pig animal genome (strain 2) has been sequenced at a 7x coverage (http://www.ensembl.org/Cavia_porcellus/Info/Index) with subsequent follow up with RNA seq analysis which potentially enables the generation of new guinea pig specific reagents. Manipulation of an infectious GPCMV BAC has allowed the preliminary study of some viral genes [19, 22–26] but, as with other animal CMV, a global knockout map has not been established unlike HCMV [27, 28].

In HCMV, a number of proteins have been identified as glycoproteins that are associated with purified virions and extra cellular dense bodies [29]. However, only six glycoproteins are essential for fibroblast cell entry in HCMV (gB, gH, gL, gM, gN, gO) and they form the glycoprotein complexes, gCI (gB), gCII (gM/gN), gcIII (gH/gL/gO) on the viral membrane [30–32]. These complexes are important neutralizing antibody targets and vaccine candidates [31, 33–36]. Clinical strains of HCMV also encode a pentameric glycoprotein complex (gH/gL/UL128/130/131) necessary for entry into epithelial and endothelial cells [29]. The locus encoding the *UL128-131* genes is unstable upon passage of clinical HCMV strains on fibroblast cells and this locus rapidly acquires point mutations or deletions with the subsequent loss of epi/endothelial viral tropism associated with the inability to form a functional complex [37]. The pentameric complex is considered an important neutralizing target for viral epi/endothelial cells and also for congenital infection, given the structure of the placenta [38]. A potential homolog pentameric complex has been identified in GPCMV and appears important for congenital infection [19, 39–41]. Recent functional studies of this complex and GPCMV epithelial tropism is reported in another paper from our laboratory, Coleman et al (paper in preparation).

The essential nature of the GPCMV glycoproteins and their role in the viral life cycle has been largely unexplored with the exception of gB, which has been investigated as a neutralizing vaccine target antigen against congenital CMV [42–45]. However, despite the ability to generate high titer antibody, it is insufficient to fully protect against congenital infection in the guinea pig model [42, 43]. Based on the viral genome sequence, GPCMV potentially encodes homologs to other HCMV glycoproteins (gH, gL, gM, gN, gO), which are encoded on genes (*GP75*, *GP115*, *GP100*, *GP73* and *GP74* respectively) which are co-linear with their counterparts in the HCMV genome. Presumably, the GPCMV homologs can generate the equivalent HCMV glycoprotein complexes to gCI, gCII and gCIII. Neutralizing antibody responses are generated in GPCMV infection [46] and passive neutralizing antibody approach has been explored as an intervention strategy against congenital infection [47]. In convalescent animals, the neutralizing antibodies are presumably directed towards the viral glycoproteins or associated homolog complexes. However, with the exception of gB, this has not been further investigated or characterized. Given the relative importance of these glycoproteins as potential candidate neutralizing antibody subunit vaccine targets, these GPCMV viral glycoproteins should be considered an important area of study for GPCMV. Therefore we analyzed the essential role of the GPCMV glycoproteins by site-specific knockout in the viral genome. Additionally, we investigated the ability of the GPCMV proteins to form homolog complexes by protein:protein interaction studies in guinea pig cells. We also investigated the immunogenicity of these complexes using convalescent sera from GPCMV infected guinea pigs and newly established ELISA assays to various glycoprotein complexes. Overall, the results of these studies indicate the similarity between HCMV and GPCMV glycoprotein complexes and strengthen the guinea pig model for congenital CMV studies and development of preclinical intervention strategies.

## Materials and Methods

### Cells, viruses and oligonucleotides

GPCMV (strain 22122, ATCC VR682), first and second generation GPCMV BAC [20, 21] derived viruses were propagated on guinea pig fibroblast lung cells (GPL; ATCC CCL 158) in F-12 medium supplemented with 10% fetal calf serum (FCS, Life Technologies), 10,000 IU of penicillin/liter, 10 mg of streptomycin/liter (Gibco-BRL), and 7.5% NaHCO3 (Gibco-BRL). The second generation GPCMV BAC encodes a truncated version of GP129 (UL128 homolog) and as such is incapable of forming a complete homolog pentameric complex (gH/gL/GP129-GP133 homolog to HCMV gH/gL/UL128-131) and lacks tropism to epithelial cells (Coleman et al., paper in preparation). In order to restore epithelial tropism to the virus, a cDNA version of the full length GP129 was introduced into the GPCMV BAC at an intergenic locus (GP25/GP26 locus) of the GPCMV genome and placed under SV40 promoter control. Virus derived from this BAC was capable of forming a pentameric complex and had restored tropism to epithelial cells. A full description of this virus, pathogenicity, tropism, congenital infection rate and pentameric complex mutant studies is the subject of a pending paper from our lab (Coleman et al.). Virus titrations were carried out on six-well plates. Plaques were stained with 10% Giemsa stain or visualized by fluorescence microscopy. All oligonucleotides were synthesized by Sigma-Genosys (The Woodlands, TX) and are listed in S1 Table. Plasmids and viral PCR products were further verified by sequencing as necessary.

### Protein structure analysis

The predicted protein sequences of the GPCMV glycoproteins were analyzed by various programs. (1) Signal peptide sequence was predicted by various on line programs: http://www.cbs.dtu.dk/services/SignalP/ [48]; http://sigpep.services.came.sbg.ac.at/signalblast.html; http://www.csbio.sjtu.edu.cn/bioinf/Signal-3L/ [49]. (2) Protein transmembrane domain predicted by http://www.cbs.dtu.dk/services/TMHMM/. (3) BLAST alignments performed on MacVector.

### Ethics

Guinea pig (Hartley) animal studies were carried out under IACUC (Texas A&M University) permit 2013#013. All study procedures were carried out in strict accordance with the recommendations in the “Guide for the Care and Use of Laboratory Animals of the National Institutes of Health.” Animals were observed daily by trained animal care staff, and animals requiring care were referred to the attending veterinarian for immediate care or euthanasia. Terminal euthanasia was carried out by lethal CO_2_ overdose followed by cervical dislocation in accordance with IACUC protocol and NIH guidelines.

All animals were verified by anti-GPCMV ELISA to be seronegative prior to their inclusion in the study. Convalescent antisera to GPCMV was generated by subcutaneous inoculation of GPCMV negative guinea pigs (n = 6) with 1x10^5 pfu salivary gland (SG) stock GPCMV (strain 22122). Seroconverted animals were verified 1 month post infection by anti-GPCMV ELISA. A booster inoculation of virus was given and final bleeds were taken at 8 weeks post initial inoculation. Serum samples were subsequently pooled for studies. Control negative sera was obtained from GPCMV negative animals, verified by ELISA, prior to initial GPCMV challenge.

### Cloning of GPCMV glycoprotein genes and generation of knockout shuttle vectors

The predicted GPCMV glycoprotein coding sequences were based on the complete viral genome sequence (Genbank accession #AB592928.1). The coding sequence co-ordinates are: *GP55* (gB) 94,164–96,869; *GP73* (gN) 117,644–118,045; *GP74* (gO) 117,992–119,104; *GP75* (gH) 119,553–121,724; *GP100* (gM) 157,482–158,531; *GP115* (gL) 180,216–180,992. All genes are encoded on the complementary strand with the exception of *GP73*. The predicted GPCMV encoded proteins were verified as homologs of HCMV glycoproteins based on the co-linear location of the GPCMV viral gene with the HCMV genome and the percentage identity of the predicted GPCMV protein with HCMV glycoproteins [9, 10, 19]. In order to carry out expression studies and a systematic knockout of the glycoprotein genes each individual GPCMV glycoprotein ORF was PCR cloned from the viral genome into plasmid vector pUC19 or pNEB193 (New England Biolabs) using specific oligonucleotide primer pairs (S1 Table) following the protocol described in McGregor et al. [24]. The following primer pair sets were used: for *GP73* oligos FGP73 & RGP73; for *GP74* oligos FGP74 & RGP74; for *GP100* oligos GP100F & GP100R; for *GP115* oligos FGPgL & RGPgL. The PCR primers also carried an additional 5’ *Eco*R I restriction enzyme site to introduce flanking *Eco*R I sites at the ends of the *GP73*, *GP74*, *GP100* and *GP115* coding sequences for ease of cloning of the PCR product. The various ORFs were cloned as *Eco*R I fragments into pNEB193 except for *GP74* which was cloned as an *Eco*R I fragment into plasmid pNEB193dSalI, which had the *Sal* I site removed from the poly-cloning region. Individual glycoprotein gene plasmid clones were verified by sequencing. The *GP75* ORF was cloned as a *Hind* III fragment using primers FGP75 and RGP75 tagged with *Hind* III sites (Table 1). The glycoprotein gene plasmids were designated: pNEBgNT for *GP73*; pNEBGP74 for *GP74*; pNEBGP100 for *GP100*; and pNEBgL for *GP115* and pNEBgH for *GP75*. Next, all of the glycoprotein coding plasmids were individually modified by the insertion of a kanamycin (Km) drug resistance marker cassette [22] to disrupt each coding sequence and enable positive selection of mutant GPCMV BAC clones. The Km cassette was inserted into a unique restriction enzyme site as close as possible to the start of the ORF for each glycoprotein gene plasmid construct. The Km cassette was amplified by PCR from plasmid template pACYC177 (New England Biolabs) using oligonuceotide primers KmF and KmR (S1 Table). These primers also introduced unique restriction sites at the 5’ & 3’ ends of the cassette to enable cassette cloning into the different target glycoprotein genes. Individual PCR products were amplified, gel purified and digested with the appropriate restriction enzyme as previously described [24]. The KmF and KmR basic primer pairs were synthesized with additional 5’ sequence for the specific restriction enzyme site. The following restriction enzyme sites were added to individual Km PCR cassettes: *Kpn* I; *EcoR* V; *Sal* I; *Nru* I; or *BamH* I. Disruption of each glycoprotein gene on each shuttle vector was carried out as follows. Plasmid pNEBgNT was digested with *Nru* I and an *Eco*R V Km cassette was inserted to disrupt the GP73 (gN) ORF at codon 71 (pNEBgNTKm). Plasmid pNEBGP74 was digested with *Sal* I and a *Sal* I Km cassette inserted to disrupt the *GP74* ORF at codon 110. Plasmid pNEBGP100 was transformed into a *dam* ^-^ strain of *E*.*coli* K12 bacteria (ER2925, New England Biolabs) and cut with *Bcl* I and a *Bam*H I Km cassette inserted to disrupt the *GP100* ORF at codon 110 (pNEBGP100km). Plasmid pNEBgL was cut with *Eco*R V to insert an *Eco*R V Km cassette to disrupt *GP115* ORF at codon 45 (pNEBgLKm). The *GP75* gene in pNEBgH was digested with *Nru* I and *Eco*R V to delete 791 bp of the coding sequence. The linearized DNA was band isolated as previously described and a *Eco*R V Km cassette inserted to disrupt the *GP75* (gH) ORF at codon 200 (pUCGP75Km). In the case of *GP55*, a shuttle vector was designed to encode the homolog gB AD-1 and trans-membrane domains of GP55 [45]. The *GP55* coding sequence (codons 400–829, genome co-ordinates 95,359–96,649c) was PCR amplified using primers FgBEc and RgBPst (S1 Table) that also introduced unique *EcoR* I and *Pst I* restriction sites at the 5’ and 3’ ends of *GP55* respectively. The *GP55* PCR product was cloned into pUC19 cut with *EcoR I* and *Pst I* to generate pUCgB. An *Eco*R V Km cassette was next introduced into pUCgB at a unique *EcoR* V site that disrupts the GP55 coding sequence at codon 528. The modified shuttle vector was designated pUCgBKm. The various glycoprotein knockout shuttle vectors (carrying kanamycin insertion markers) were verified by sequencing and subsequently used to create individual glycoprotein gene mutants on the GPCMV BAC in bacteria. Modified plasmids encoding Km cassettes are described in S1 Fig.

**Table 1:** GPCMV Glycoprotein Genes, predicted size, homology and knockout site.

GPCMV Gene (co-ordinates)	Glycoprotein (predicted size)	Signal peptide predicted	Glycosylation Sites	% Identity with HCMV (BLAST)	Site of ORF Knockout
*GP55* (c94164-96869)	gB (901 aa/ 102.2 kDa)	yes	32 (O-linked) 15 (N-linked)	45%	codon 528 insertion
*GP73* (117644–118045)	gN (134 aa/ 14 kDa)	yes	17 (O-linked) 2 (N-linked)	44%	codon 71 insertion
*GP74* (c117992-119105)	gO (370 aa / 41.8 kDa)		13(O-linked) 13(N-linked)	27%	codon 110 insertion
*GP75* (c119553-121724)	gH (724 aa/ 81.8 kDa)	yes	10 (O-linked) 9 (N-linked)	29%	codon 200 insertion/del
*GP100* (c157482-158531)	gM (349 aa/ 39.7 kDa)	yes	0 (O-linked) 2 (N-linked)	52%	codon 170 insertion
*GP115* (c180216-180992)	gL (258 aa/ 29.7 kDa)	yes	3 (O-linked) 3 (N-linked)	42%	codon 45 insertion

c = complement DNA strand coding, aa = amino acids.

Co-ordinates based on complete GPCMV (22122 strain) sequence (GenBank: AB592928.1); Kanai et al. [10]. Percentage identity determined by BLAST analysis of GPCMV against HCMV Towne strain. Predicted protein size is based on complete protein predicted sequence and calculated using MacVector.

Signal peptide sequence predicted by web based programs. See S4 Fig

Post translational glycosylation predicted based on web programs: NetOGlyc 4.0 Server (http://www.cbs.dtu.dk/services/NetOGlyc/) for O-glycosylation; and NetNGlyc 1.0 Server (http://www.cbs.dtu.dk/services/NetNGlyc/) for N- glycosylation. Total predicted number of N-glycosylation or O-glycosylation sites per glycoprotein are indicated.

Insertion site of the kanamycin (Km) cassette to knockout the various coding sequences was carried out using convenient restriction sites (see materials and methods). The insertion of the Km cassette disrupts the ORF at the specified codon.

#### Construction of GPCMV gB mammalian expression vector

First a low copy number expression plasmid was generated on the backbone of pACYC177 (New England Biolabs) to enable stable maintenance of the *GP55* ORF. The HCMV MIE promoter and polycloning linker sequence was isolated from pcDNA3 (Invitrogen) as a *Bgl* II/ *EcoR* V fragment and cloned into pACYC177 isolated as a *BamH* I/*Nru* I fragment to remove the kanamycin cassette to generate pACYCIE. The SV40 polyA cassette was then subcloned as an *Eco*R I fragment from an existing clone into pACYCIE cut with *EcoR* I to generate pACYCIESV. A full length gB expression construct was also generated by PCR cloning the complete ORF as a *Bgl* II fragment using forward primer Fgp55Bgl and reverse primer Rgp55fullBgl (S1 Table). The full length gB was cloned into pACYCIESV to generate pACYCIEgB. The gB ORF was sequenced to verify integrity of the gB.

#### Construction of GPCMV gH, gL and gO glycoprotein tagged mammalian expression vectors

The gH (*GP75*), gL (*GP115*) and gO (*GP74*) ORFs were additionally separately cloned into expression vectors that also tagged the C-terminal domain for easy detection of the recombinant protein in transfected cells. For GFP tagged gH, the *GP75* ORF (missing the stop codon) was PCR amplified as a *Bam*H I fragment using primers FgHBm and RgHBmNostop (S1 Table) and cloned inframe into GFP fusion expression vector pAcGFP-N1 (Clontech) cut with *Bam*H I. This introduced a GFP tag in-frame into the C-terminal domain of the gH ORF and placed the *GP75* under a HCMV MIE promoter control. This modified plasmid was designated pAcGFPNgH.

For mCherry tagged gL, the *GP115* ORF (missing the stop codon) was PCR amplified as a *Hind* III fragment using primers FgLHd and RgLHdNostop (S1 Table) and cloned inframe into mCherry fusion expression vector pmCherry-N1 (Clontech) cut with *Hind* III. This introduced a mCherry tag in-frame into the C-terminal domain of the gL ORF and placed the *GP115* under a HCMV MIE promoter control. This modified plasmid was designated pmCherryNgL.

For FLAG tagged gO, the *GP74* ORF was PCR amplified without a stop codon using primers FGP74EcV and RGP74XhoNostop (S1 Table) digested with *EcoR* V and *Hind* III and cloned inframe into the C-terminal FLAG epitope tag expression vector pCMV-3TAG-8 (Stratagene) as a *EcoR* V and *Xho* I fragment. This introduced an in frame 3x FLAG tag epitope tag into the C-terminal domain of gO and placed the gene under HCMV MIE promoter control. The modified plasmid was designated pGP74TAG8. A mutant version of the GP74 ORF was generated synthetically (DNA2.0) where all potential N-linked glycosylation sites (13 identified) were modified by changing the codon sequence of the putative N-linked glycosylation sites (NXT to NXA) to prevent glycosylation (see results section). The C-terminus of the GP74 mutant ORF was also 3xFLAG tagged similar to wild type GP74. The *GP74* mutant ORF (designated GP74def) was placed under HCMV MIE promoter control in a mammalian expression plasmid pJ603 (DNA2.0 Inc.).

#### Construction of GPCMV gM and gN glycoprotein tagged mammalian expression vectors

The gM (*GP100*) and gN (*GP73*) ORFs were separately cloned into expression vectors that also tagged the C-terminal domain (GFP for gM, mCherry or FLAG for gN). For GFP tagged gM, the *GP100* ORF was PCR cloned as a *BamH* I (5’) *EcoR* I (3’) fragment lacking a stop codon into pAcGFP-N1 (Clontech) using primers gMTagF and gMTagR. For mCherry tagged gN, The *GP73* ORF minus a stop codon was similarly PCR cloned as a *Bam*H I (5’) *Eco*R I (3’) fragment into pmCherry-N1 (Clontech) using primers gNTagF and gNTagR. For FLAG tagged gN, both full length (codons 1–132) and a N terminal truncated (codons 40–132) *GP73* ORF were PCR cloned separately (lacking a stop codon) into the expression vector pCMV3Tag8 (Agilent technologies Inc.) as *BamH* I / *Hind* III fragments to C-terminal epitope tag the ORFs using forward primers FGP73BmShort for truncated GP73 or FGP73BmFull for full length GP73 plus reverse primer RGP73nostopHd (S1 Table). Recombinant plasmids were designated pgN(f)FLAG and pgN(s)FLAG for full length and truncated expression plasmids respectively.

### Generation of gene knockout GPCMV BACmids and analysis of GPCMV BAC mutants

An inducible ET recombination system (GeneBridges) was introduced into DH10B bacterial cells containing a first or second generation GPCMV BAC plasmid [20, 21] using a protocol previously described [24]. Individual GPCMV glycoprotein knockout targeting shuttle vectors were linearized with a unique restriction enzyme cutting outside the glycoprotein gene flanking sequence or alternatively the kanamycin cassette and glycoprotein gene flanking coding sequence were amplified by PCR using gene specific primers as described in S1 Table. Linearized DNA plasmids or PCR products were band isolated by agarose gel electrophoresis and recovered by gene clean kit (MP Biomedical). Concentrations of DNA were modified to introduce 1μg of linear DNA into each transformation reaction via electroporation [24]. Recombinant bacterial colonies of GPCMV BAC glycoprotein knockout mutants were isolated by chloramphenicol (12.5 μg/ml) and kanamycin (20 μg/ml) antibiotic selection in LB agar bacterial Petri dishes. Bacterial plates were initially incubated at 39°C to remove the ts ET recombination plasmid (Genebridges). Mutant GPCMV BAC DNA purified by maxiprep kit (Qiagen) were analyzed by separate *Eco*R I and *Hind* III restriction digestions to verify the accuracy of the predicted genome configuration after mutation [20, 21]. Insertion of the Km drug resistance cassette into the viral genome introduced a novel *Hind* III restriction enzyme site at the site of mutation to enable verification of locus modification. Specific glycoprotein gene modifications were confirmed by comparative PCR analysis between wild type and mutant GPCMV BACs using common flanking primers for each gene (S1 Table). PCR reactions were carried out using conditions described in McGregor et al. [24] except the extension time at 72°C was modified based on the size of each gene (based on 30 sec extension per 500 bases). The gene knockout for mutants was further verified by sequencing of the PCR product.

### GPCMV glycoprotein knockout & BAC mutagenesis characterization results

GPCMV glycoproteins genes were individually knocked out by targeted mutagenesis of the GPCMV BAC in bacteria using shuttle vectors carrying a Km drug resistance marker to disrupt each ORF. The specific site of disruption for each glycoprotein is summarized in Table 1. Targeted recombination knockout of each glycoprotein gene on the GPCMV genome was performed in separate transformation reactions in both first generation [20] and second generation [21] GPCMV BACs and gene knockouts were selected by insertion of the Km cassette into the GPCMV BAC genome. S1 Fig shows the location of the genes in the viral genome as well as the restriction enzyme profile analysis of the various mutant GPCMV BAC clones. Analysis shown is for the mutagenesis of the second generation GPCMV BAC [21]. Insertion of the Km cassette also introduced a new *Hind* III site encoded in the Km ORF. Modified GPCMV genomes were analyzed separately by *Eco*R I and *Hind* III restriction enzyme profile analysis. In an effort to limit redundancy the profiles shown for each mutant are either *Hind* III or *EcoR* I analysis. Additionally, two clonal mutants were generated for each knockout but only one is described. Comparative restriction fragment profiles of wild type and mutant GPCMV BAC genomes correctly demonstrated specific sub-genomic fragment modification for all mutants. Original designated GPCMV restriction fragment band nomenclature described by Gao and Isom [50] is used to identify specific band shifts. *Eco*R I profiles are shown for *GP73*, *GP74*, *GP75*, *GP100* and *GP115* knockouts. *Hind* III profile is shown for *GP55* knockout. The *GP73* (117,644–118,045) ORF is encoded in the *Eco*R I ‘C’ fragment (18,026bp) and was modified by a 1.1 kb Km marker insertion. This resulted in the modified ‘C’ fragment shifting and partially overlapping with the *Eco*R I ‘B’ fragment (19,766bp), see S1(iii) Fig. Similarly, the GP74 (117,992–119,1104, complementary strand) ORF is encoded on the *Eco*R I ‘C’ fragment with a 1.1 kb shift in size because of the Km marker insertion, see S1(iv) Fig. The GP75 (119,553–121,724, complementary strand) ORF is encoded in both the *Eco*R I ‘C’ and ‘B’ fragments. An internal deletion within the ORF during mutagenesis (S3 Fig) resulted in the loss of the separate bands in the mutant and a fused modified ‘C+B’ bands which has a 1.1 kb Km cassette insertion and the shift with fused bands results in an overlap with the *Eco*RI ‘A+y’ band (38,915bp), see S1(v) Fig. The GP100 (157482–158531, complementary strand) ORF is in the *Eco*R I ‘E’ band (11,210bp) and a 1.1kb insertion results in a shift in the band to 12.3 kb which partially overlaps with the ‘D’ fragment (12,061bp), see S1 Fig. The *GP115* (180,216–180,992, complementary strands) is encoded in the ‘F’ fragment (174,634–184,516) and 1.1 kb insertion modifies the subgenomic fragment from 9,882bp to 10,982bp, see S1(vii) Fig. The GP55 (94,164–96,869, complementary strand) ORF encoded in the *Hind* III ‘K’ fragment (8,056bp) was modified by Km cassette insertion which increased the size of the fragment by 700bp because of the presence of a *Hind* III site within the Km cassette, see S1(ii) Fig.

The modified loci for the GPCMV glycoprotein gene mutants were also confirmed by specific PCR analysis of wild type and disrupted glycoprotein genes using common flanking primers for each gene (S1 Table). The predicted sizes of PCR products for both wild type and mutated glycoprotein genes are shown in S2 Fig using common flanking primer pairs described in S1 Table. S3 Fig shows the actual PCR results for the GPCMV glycoprotein BAC wild type and mutant loci using the common flanking PCR primer pair for each gene which produced results as expected.

### Recombinant Adenovirus vectors

Recombinant defective adenoviruses (serotype 5) encoding either GFP tagged gH or mCherry tagged gL or gB were generated as high titer stocks by Welgen Inc. on HEK293 cells. The C-terminal tagged ORFs from plasmids pAcGFPNgH and pmCherryNgL or the non-tagged complete ORF of gB from pACYCIEgB (described above) were each placed under HCMV MIE enhancer promoter control in the E1 locus of the defective Ad vectors using a IE1 shuttle vector (Welgen Inc.) to generate recombinant defective adenoviruses designated AdgHGFP, AdgLmCherry and AdgB respectively. A defective Ad vector encoding GFP (AdGFP) was also used in control expression studies.

### Generation of mutant virus and rescue of knockout mutants

For generation of recombinant viruses, large-scale GPCMV BAC DNA was purified from *E*. *coli* DH10B strain using a maxi plasmid kit (Qiagen). BAC DNA was transfected onto GPL cells in six well dishes using Lipofectamine 2000 (Invitrogen) as previously described [51]. GPCMV BAC transfections were carried out with two independent clones for each gene knockout. Transfections were followed for at least 3–4 weeks for the production of viral plaques. GFP positive viral plaques were detected via microscopy [51]. Non-infectious mutants produced only single GFP positive cells that did not progress to viral plaques. GPCMV mutant BAC transfections were carried out multiple times (minimum of 6 times) for each clone.

For glycoprotein gene knockout mutants, each mutant was rescued back to wild type phenotype by co-transfection with the appropriate rescue plasmid. Rescue plasmids were generate as full length glycoprotein genes cloned individually into pUC19, pNEB193 (New England Biolabs) or pLITMUS28 (New England Biolabs). Rescue plasmids provided sufficient flanking homologous recombination sequence around the site of Km insertion on the mutated GPCMV BAC to enable efficient generation of rescue virus. All rescue viruses (*GP55*, *GP73*, *GP74*, *GP75*, *GP100* and *GP115*) were generated as previously described [22, 24]. Additionally, gH knockout derived virus was supported on a complementing GPL cell line which expressed gH in *trans*. The GP75 ORF was placed under HCMV MIE promoter control in an existing sleeping beauty construct [52] and cell line generated by transposition of GPL cells and selection under neomycin (G418) resistance at a selection concentration of 400 μg/ml. Complemented or rescued virus was determined by the spread of GFP tagged virus on the various cell monolayers. Specific rescue was confirmed by DNA extraction from virus infected cells and PCR analysis using common flanking gene specific primers for appropriate loci (S1 Table) as previously described [24].

### RT-PCR

Time point samples were taken from wild type GPCMV infected GPL cells in a six well dish (moi = 1 pfu/cell) at 0, 4, 8, 16, 24, 48 hr post infection. RT-PCR was performed essentially as described in McGregor et al. [51]. RT-PCR reactions were performed for GPCMV glycoprotein genes *GP73*, *GP74*, *GP100*, and control *GAPDH* using primers described in S1 Table at late stage infection. In another duplicate study, chemical inhibitors (cycloheximide at 100μg/ml or phosphonoacetic acid at 200μg/ml) were included to identify the different classes of transcripts (IE, E or L). Inhibitors were used as described by Yin et al. [53]. RT-PCR was also performed on the major immediate early transcripts for GPCMV *IE2*, *GP122* unique exon, (data not shown) using primers previously described [51].

### Immunodetection assays

#### Western blot analysis

Western blot and immunofluorescence assays were carried out as previously described [24, 51]. However, convalescent GPCMV positive sera was initially pre-absorbed with acetone:methanol (1:1 ratio) fixed uninfected fibroblast cells to remove background non-specific binding antibodies prior to use in immunological assays

### ELISAs

#### Anti-GPCMV ELISA

An in house anti-GPCMV IgG ELISA was carried out as previously described [54]. Briefly, GPL cells infected with wild type GPCMV (strain 22122) were harvested, washed in 1X PBS twice, sonicated (3 cycles of 5s pulses, 50% amp; Sonicator Q500) then centrifuged (10,000 x g for 20mins 4°C) to collect cleared cell lysate as positive coating antigen (Ag+). In parallel, uninfected cells were similarly prepared as negative coating antigen (Ag-). A BCA protein assay (Pierce) was performed on clarified supernatant to determine the protein concentration according to manufacturer’s instruction. Coating antigens were titrated to determine the optimal coating concentration. MaxiSorp ELISA plates (NUNC) were coated with 0.25μg of either Ag+ or Ag- preparations diluted in carbonate coating buffer overnight at 4°C, washed in PBST then blocked with 2% nonfat dry milk. Test sera were diluted in blocking buffer from 1:80 to 1:5120 in doubling dilutions, incubated for 2 hours at 37°C and then reacted with anti-Guinea Pig IgG peroxidase antibody (Sigma) diluted (1:1000) in blocking buffer for an additional 1 hour at 37°C before reacting with TMB membrane peroxidase substrate (KPL). Net OD (absorbance 450nm) was attained by subtracting OD of Ag- from OD of Ag+. ELISA reactivity was considered positive if the net OD was greater than or equal to 0.2 as determined by GPCMV negative serum. A commercial anti-GPCMV ELISA kit (XpressBio) was also used to compare sensitivities to our in house assay following manufacturer’s protocol.

#### Anti-GPCMV glycoprotein complex specific ELISA

For specific glycoprotein complex ELISA (gB, gM/gN, or gH/gL), GPL cells were transfected with expression plasmids encoding glycoprotein(s) or control GFP (pAcGFP-N1, Clontech) following previously described transfection protocol [24]. As described above for anti-GPCMV ELISA, transfected cells were harvested, washed, and sonicated. Results were positive when the OD of Ag- subtracted from the OD of Ag+ was greater than or equal to 0.2 as determined by GPCMV negative sera.

#### Anti-Glycoprotein B depleted serum ELISA

Anti-GPCMV and anti-gB ELISA were performed on gB antibody depleted GPCMV convalescent pooled serum as described above. For anti-gB depletion, HEK 293 cells were transduced with recombinant defective adenovirus expressing gB (moi = 10 TDU/cell). Cells were harvested, washed twice with PBS, then fixed with 1:1 ratio acetone:methanol fixation mixture for 20 mins at -20°C. Fixed cells were pelleted then resuspended in 500μl of PBS + 1% tween 20. Equal volume of convalescent serum was used for preabsorption overnight at 4°C in a tube rotator. Cells were centrifuged at 10,000 x g for 20 mins at 4°C to pellet, serum collected then stored at -80°C until needed.

### Immunoprecipitation assay

Immunoprecipitation (IP) assays were carried out on plasmid transfected or recombinant Ad transduced fibroblast cells using commercial GFP-trap reagent (ChromoTek) following manufacturer’s protocol and inclusion of protease inhibitor cocktail (Pierce) in cell lysates. Samples were subsequently analyzed by SDS-PAGE (4–20% gradient gel) and western blot using specific anti-epitope tag antibodies: HA (Novus Biologicals); FLAG (Novus Biological); GFP (Santa Cruz); Myc-c (Novus Biologicals); and mCherry (Clontech). Appropriate secondary anti-mouse or anti-rabbit HRP conjugate (Cell Signaling Technology) were also used following standard western blot protocol.

### GPCMV Neutralization assay

GPCMV neutralization assays were conducted using convalescent GPCMV positive sera. Serially diluted guinea pig serum was incubated with approximately 50 pfu of GFP positive GPCMV, vAM403 [20], in media containing 0.5% rabbit complement (Equitech Bio, Kerrville, TX) for 60 minutes at 37°C. Each dilution was done in duplicates and virus only was used as control. To show neutralization specificity, monoclonal antibody 29–29 was used at 1:500 dilution in place of anti-GPCMV sera [44]. The neutralization reactions were added to wells of a 12 well-plate containing confluent monolayers of GPL cells and incubated for 2 hours. The wells were washed twice with PBS before complete F12- media was added and incubated for 3–5 days at 37°C. The plates were fixed with 4% paraformaldehyde and GFP positive plaques counted using a fluorescent microscope. Control assays were also performed with GPCMV negative sera which did not inhibit virus infection at dilutions used.

## Results

### GPCMV Glycoprotein gene expression

Based on the GPCMV genome sequence [10], the GPCMV glycoproteins associated with the homolog complexes in HCMV are predicted to be encoded by the GPCMV genes *GP55* (gB), *GP73* (gN), *GP74* (gO), *GP75* (gH), *GP100* (gM), *GP115* (gL). Analysis of the predicted proteins (http://www.cbs.dtu.dk/services/SignalP/) indicated potential N-terminal signal leader peptide sequence of 23 and 24 amino acids in gB and gH respectively (S4 Fig). N-terminal signal peptide sequences were also predicted (http://sigpep.services.came.sbg.ac.at/signalblast.html) for gM (26 amino acids), gL (31 amino acids) and gN (44 amino acids), see S4 Fig The gO homolog protein had insufficient conserved sequence (based on results from analysis programs used) to fully predict a signal peptide sequence. However, the inability to detect a N-terminal epitope/GFP tagged glycoprotein in transient expression assays (data not shown) in contrast to successful detection of C-terminal epitope tagged proteins in transient expression studies (see individual glycoprotein expression results) would suggest that all the GPCMV glycoproteins have N-terminal signal sequences. Previous transient expression studies with other N-terminal tagged, epitope (FLAG) or GFP, GPCMV proteins demonstrate the success of this approach for detection of proteins that lack an N-terminal signal peptide sequence. These previous GPCMV protein expression studies included: GP35 (UL35 homolog), GP44 (viral DNA polymerase subunit), GP82 (pp71 homolog), GP83 (pp65 homolog), GP84 (UL84 homolog), GP97 (viral kinase) [22–24, 55]. The size of the predicted GPCMV glycoproteins and the percentage identity to HCMV glycoproteins by BLAST analysis is indicated in Table 1. The gB (gCI homolog complex), gM and gN (gCII homolog complex) exhibit the highest identity to HCMV proteins. All of the viral glycoproteins have additional potential post-translational modification by glycosylation (O or N linked glycosylation) and the total number of potential sites are indicated in Table 1 and discussed in later sections. Most notably, the gO protein homolog encoded by *GP74* is predicted to be heavily N-glycosylated as is the case for HCMV gO and the potential glycosylation sites are conserved between HCMV and GPCMV. This is discussed in more detail in a later section on the gH/gL/gO complex.

**Fig 1:**
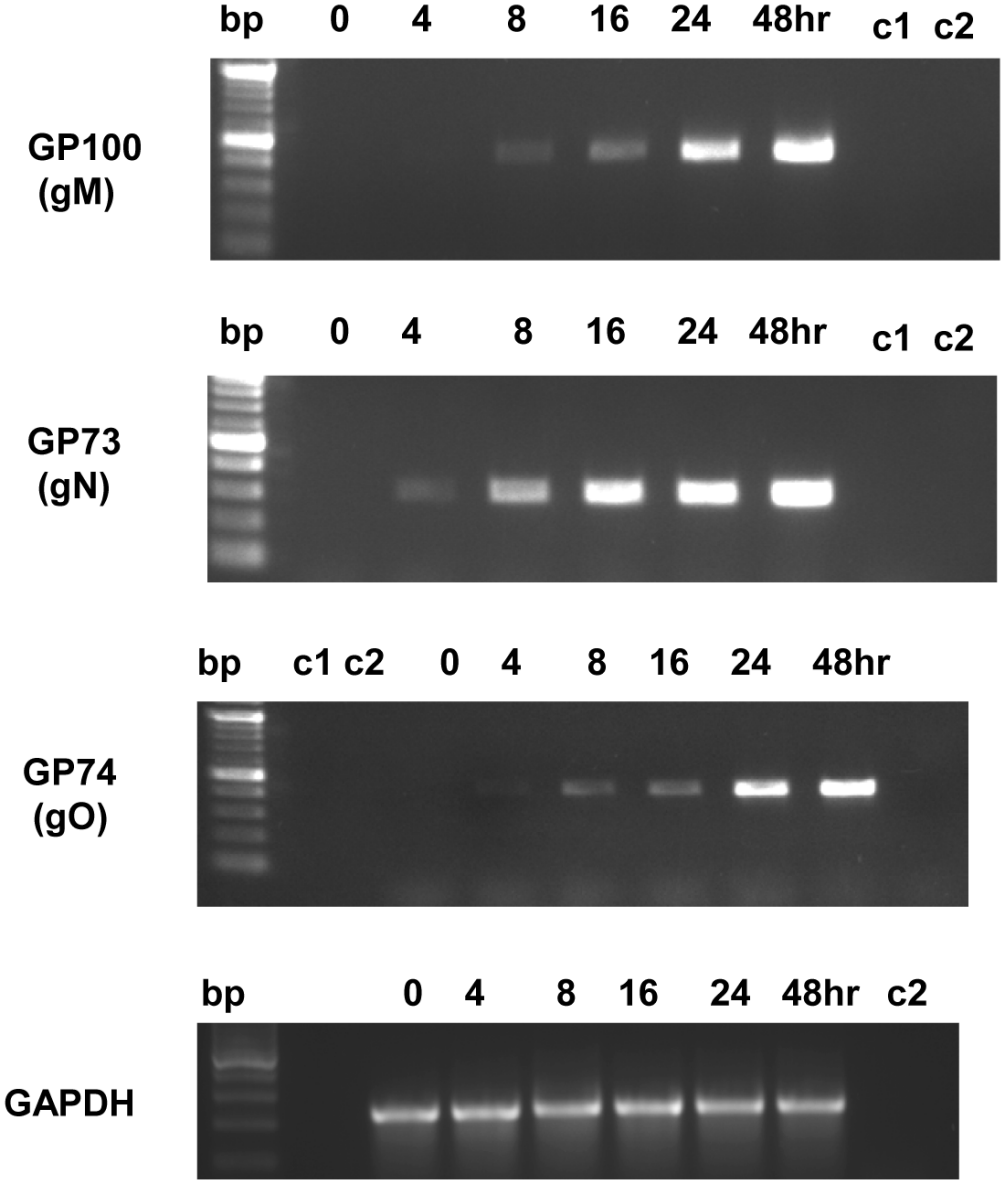
Electrophoresis of GPCMV glycoprotein gene expression by RT-PCR assay. Agarose gel analysis of RT-PCR products. Expression of viral glycoprotein genes *GP73* (gN), *GP74* (gO) and *GP100* (gM) was investigated at various time points post infection. Input virus was wild type GPCMV (strain 22122) on GPL cells (moi = 1 pfu/cell). RT-PCR was performed as described in materials and methods. RT-PCR analysis of time point samples 0, 4, 8, 16, 24, 48 hr post infection indicated. Controls: C1, no template; C2, infected cell lysate no reverse transcriptase stage. *GAPDH* control RT-PCR for all time samples.

Expression kinetics of the GPCMV gB, gH and gL genes (*GP55*, *GP75* and *GP115* respectively) have been previously described.[45, 56, 57]. Transcription from genes *GP73*, *GP74* and *GP100* (encoding gN, gO and gM respectively) were analyzed by RT-PCR at various time points post infection (input virus moi = 1pfu/cell). The time course assay (Fig 1) indicated that the *GP73*, *GP74* and *GP100* genes were expressed under similar kinetics based on comparison with genes from different classes of expression: immediate early, IE, (*GP122*); early, E, (*GP54*); and late, L, (*GP83*) [51, 58, 59]. The glycoprotein genes (*GP73*, *GP74* and *GP100*) are expressed as early/late genes. The use of transcription chemical inhibitors (cycloheximide and phosphonoacetic acid) more precisely place *GP73* (gN), *GP74* (gO) and *GP100* (gM) as late transcripts (S5 Fig) as transcripts failed to be detected in the presence of inhibitors. As a positive control, the GPCMV IE2 transcript (GP122) could be detected in these samples (S5 Fig) which demonstrated the presence of viral transcripts and integrity of the samples.

### Transient expression studies of GPCMV homolog glycoproteins

In order to evaluate cellular location and interactions of the GPCMV glycoproteins, the various ORFs were cloned into commercial mammalian expression vectors and epitope tagged at the C-terminus. Construction of mammalian expression vectors encoding the GPCMV glycoprotein ORFs was as described in materials and methods. Transient expression studies were carried out on GPL cells and subsequently analyzed by: western blot, to verify protein size; immunofluorescence, for cellular co-localization of proteins; immunoprecipitation assay, to demonstrate specific protein:protein interactions.

### GPCMV gM (GP100)/gN (GP73) studies

**Fig 2:**
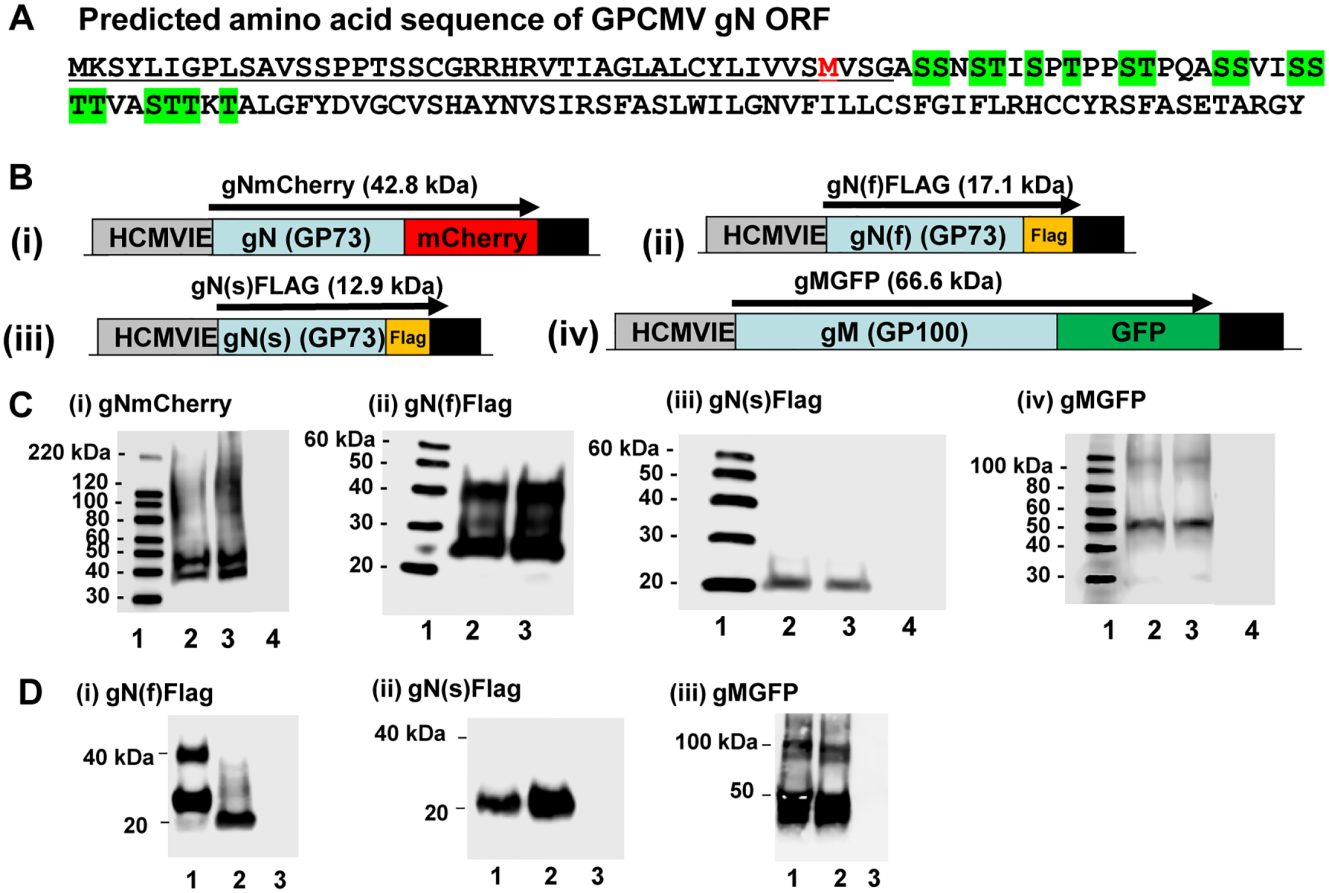
Transient expression of GPCMV gM and gN homologs and analysis of tagged proteins by western blot. **A**. Predicted amino acid sequence for GP73 (gN) with potential glycosylation sites shaded in green. A truncated version of gN was generated by deletion of the first 40 codons, which included the majority of the predicted signal peptide sequence (underlined, see also S4 Fig). The truncated gN(s) initiated from the first internal methionine (shaded in red). **B**. Figure of gN and gM expression constructs. Full length gN tagged with C-terminal mCherry was designated gNmCherry, B(i). Full length gN with C-terminal FLAG epitope tag was designated gN(f), B(ii), and truncated gN designated gN(s), B(iii). Full length gM was C-terminal tagged with GFP and designated gMGFP, B(iv). Size of predicted MW of tagged proteins indicated (kDa). **C**. Western blots were performed on transient plasmid expression of gN and gM tagged proteins in GPL cells. C(i) gNmCherry expression detected using anti-mCherry antibody. Lanes: (1) kDa ladder; (2) and (3) gNmcherry; (4) mock untransfected GPL cell lysate. C(ii) gN(f)FLAG expression detected using anti-FLAG antibody. Lanes: (1) kDa ladder; (2) and (3) gN(f)FLAG. C(iii) gN(s)FLAG expression detected using anti-FLAG antibody. Lanes: (1) kDa ladder; (2) and (3) gN(s)FLAG (4) mock untransfected GPL cell lysate. C(iv) gMGFP expression detected using anti-GFP antibody. Lanes: (1) kDa ladder; (2) and (3) gM; (4) mock untransfected GPL cell lysate. **D**. gN and gM expression in the presence or absence of glycosylation inhibitor. D(i) Western blot of gN(f)FLAG in the presence of tunicamycin (lane 2) or absence (lane 1). D(ii) Western blot of gN(s)FLAG in the presence of tunicamycin (lane 2) or absence (lane 1). D(iii) Western blot of gMGFP in the presence of tunicamycin (lane 2) or absence (lane 1). Control mock untransfected GPL cell lysate lane 3 D(i)-(iii).

In HCMV, gM/gN proteins form a glycoprotein complex [60] and potentially GPCMV encodes a homolog complex. In GPCMV, gM is predicted to be a type III membrane protein with 8 possible membrane spanning domains and GPCMV gN is predicted to be a type I membrane protein with two potential transmembrane domains which is similar to HCMV. S6 Fig compares the predicted transmembrane domains of gM and gN in GPCMV and HCMV which show similarity in location of predicted transmembrane domains respectively. Expression plasmids for gN (GP73 mCherry or FLAG C-terminal tagged) and gM (GP100 C-terminal GFP tagged) were used to transiently express proteins individually (Fig 2). As with HCMV, GPCMV gN is predicted to be post-translationally modified by both O and N-linked glycosylation (Table 1) and two gN bands were detected with higher than expected molecular weight for both gNmCherry and gNFLAG. The predicted sizes for gN tagged proteins are shown in Fig 2, gNmCherry (42.8 kDa) and gN(f)FLAG (17.1 kDa). Two different molecular weight species were detected for each tagged gN protein: approximately 37 and 45 kDa (gNmCherry); and approximately 25 and 40 kDa (gN(f)FLAG). In order to demonstrate that the higher molecular weight protein was associated with post-translational modification of gN a N-terminal truncated gN (gN(s)FLAG) was generated. This ORF, which lacked the first 40 amino acids (as well as a complete predicted signal peptide sequence (S4 Fig), underlined, Fig 2A), initiated at the first internal methionine (codon 41 highlighted in red, Fig 2A). The full length gN (gN(f)FLAG) with an intact predicted signal peptide leader sequence (Fig 2A and S4 Fig) could undergo post translation modification. In contrast, the truncated gN(s) which lacked the leader N-terminal sequence would be impaired for post-translational modification. Western blot assays of plasmid transfected cell lysates detected a single species of protein for the truncated gN(s)FLAG of approximately 20 kDa in size, see Fig 2C(iii). This contrasted with the full length gN (gN(f)FLAG), where two species of gN protein could be detected (25 and 40 kDa), Fig 2C(ii). This indicated that the N-terminal end of the gN protein contained a peptide leader sequence (predicted size of 44 amino acids, S4 Fig) that was most likely necessary for post-translational modification of gN. Importantly, all of the potential glycosylation sites of full length gN were retained in the truncated gN(s) construct (Fig 2A). Tunicamycin (N-glycosylation inhibitor) was included in the transient expression of full length and truncated FLAG tagged gN proteins to further verify post translational modification. In the presence of tunicamycin, the full length gN (gN(f)FLAG) was detected as a single molecular weight species of approximately 20kDa (Fig 2D(i)), whereas the size of the truncated gN was unaffected by the presence of the inhibitor. We concluded that the higher molecular weight protein was a result of post-translation modification which was most likely N-glycosylation. However, even in the presence of tunicamycin, the truncated gN protein had a higher molecular weight than expected and potentially this might be due to predicted O-glycosylation of gN but this awaits further investigation.

In contrast to gN, the gM (GP100) protein is not predicted to be as extensively glycosylated with two N-glycosylation sites predicted (see Table 1). Western blot analysis indicated that the gMGFP fusion protein was smaller than expected in size, approximately 55 kDa compared to the predicted size of 66.6 kDa (Fig 2B and 2C(iv)). HCMV gM [60] encodes a signal peptide leader sequence and a signal sequence is predicted for GPCMV gM (S4 Fig). Presumably, cleavage of the predicted signal peptide sequence (26 amino acids) accounted for the difference in size of the detected protein. Inclusion of tunicamycin in the transient expression assay of gMGFP did not appear to have any effect on the size of the detected protein (Fig 2D(iii)) which supported the assumption that gM is not extensively post-translationally modified by glycosylation. In gMGFP western blots, a fainter higher molecular weight species was detected at about 100 kDa which might represent a non-fully denatured form of the protein. It should be noted that gM samples in Laemmli denaturing loading reagent were heated at 37°C for 5 minutes prior to loading on a SDS-PAGE gel as opposed to the normal procedure of 95°C for 5 minutes as we found that samples treated at high temperature became partially insoluble and did not enter the gel and instead were retained in the wells of the gel. Presumably, the predicted 8 transmembrane structure of the protein (S6 Fig) accounted for the technical difficulty associated with analysis of this protein by SDS-PAGE. Future experiments with denaturing urea conditions might help further improve protein resolution.

**Fig 3:**
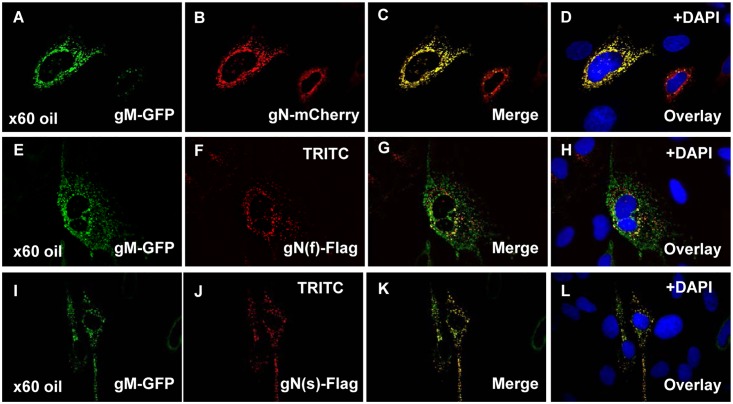
Transient co-expression of GPCMV gM and gN. Tagged versions of gN and gM were transiently co-expressed in GPL cells and cellular localization patterns investigated by immunofluorescence or autofluorescence assay. Panels **A-D**, gMGFP and gNmCherry co-localization studies. A and B show gM and gN separately within the same cell. C is the merged image for A and B. D is the overlay of C with DAPI stain to indicate location of the nucleus. **E-H**, gMGFP and gN(f)FLAG co-localization with G merged image for panels E and F. H the overlay for DAPI stain with merged image G. **I-L**, gMGFP and gN(s)FLAG co-localization with K merged image for I and J. L overlay with DAPI staining.

**Fig 4:**
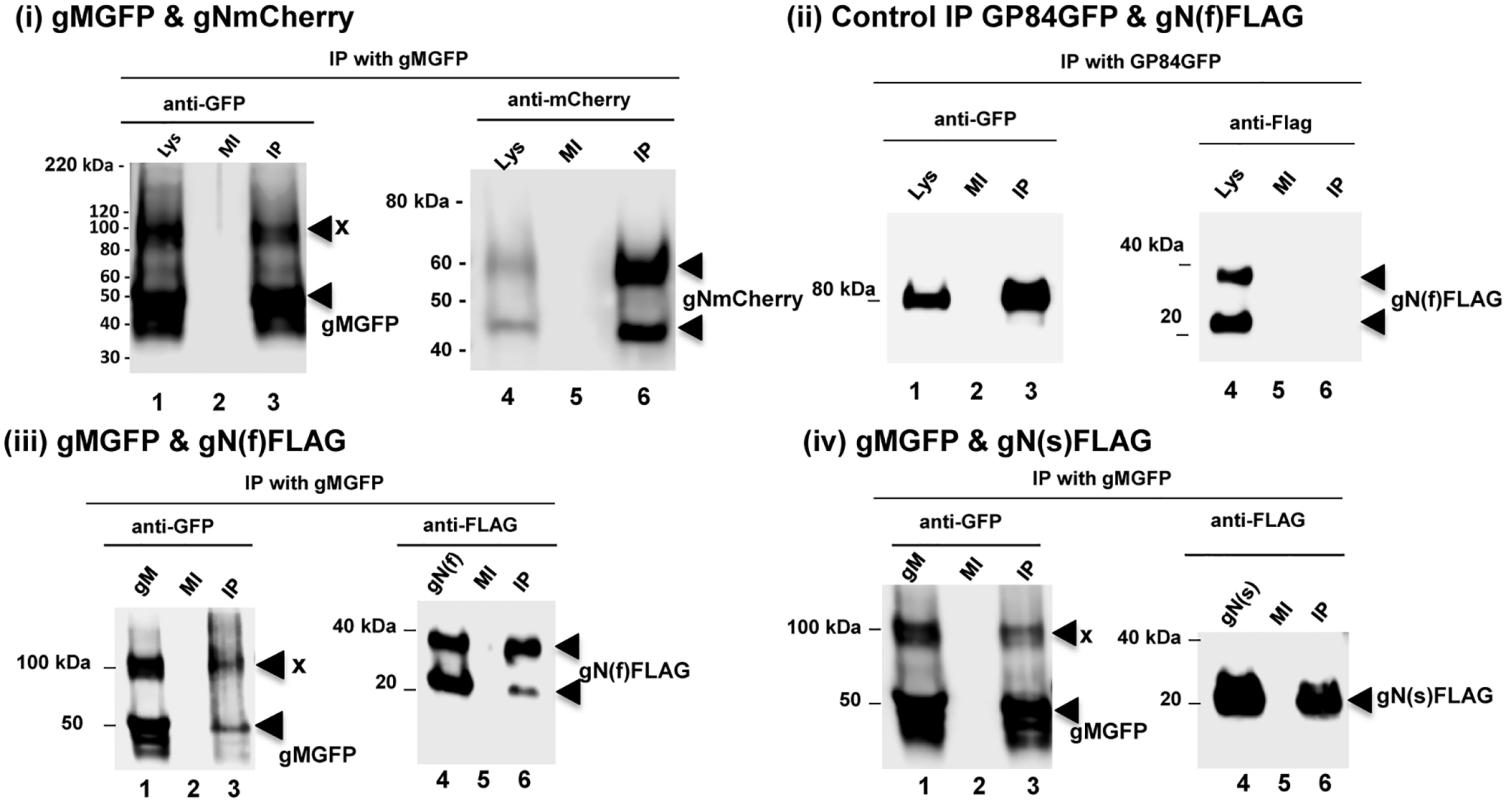
GPCMV gM /gN complex formation and immunoprecipitation (IP) assays. All IPs were performed with GFP-Trap (ChromoTek) as described in materials and methods to immunopreciptiate proteins that interacted with gMGFP or GFP control. **(i)** gMGFP and gNmCherry co-expression and IP. Lanes 1 and 4 are total cell lysate of gMGFP and gNmCherry, respectively. Lanes 3 and 6, IP reactions. Lanes 2 and 5, control mock (MI) cell lysate. For gM detection, anti-GFP antibody (lanes 1–3). For gN detection, anti-mCherry antibody (lanes 4–6). **(ii)** Control GFP IP. GP84 protein tagged with GFP [51] was co-expressed with gN(f)FLAG. Lanes 1 and 4 total cell lysate for GP84GFP and gN(f)FLAG respectively. Lanes 3 and 6, IP reactions for GP84GFP and gN(f)FLAG transfected cells. Lanes 2 and 5 mock control (MI). **(iii)** gMGFP and gN(f)FLAG co-expression and IP. Lanes, 1 and 4 are total cell lysates of gMGFP and gN(f)FLAG transfected cells respectively. Lanes 3 and 6, IP reactions for gMGFP and gN(f)FLAG transfected cells. Lanes 2 and 5 mock (MI) control cell lysate. 6. Detection for gN(f)FLAG by anti-FLAG antibody. **(iv)**. gMGFP and gN(s)FLAG co-expression and IP. Lanes, 1 and 4 are total cell lysate of gMGFP and gN(s)FLAG transfected cells respectively. Lanes 3 and 6, IP reactions for gMGFP and gN(s)FLAG transfected cells. Lanes 2 and 5, mock control (MI). Detection for gN(s)FLAG by anti-FLAG antibody. Specific protein bands are indicated by an arrow. In gMGFP expressing cells a second higher MW protein (100 kDa) was detected and labelled x. All gels (4–20%) SDS-PAGE included a lane for a kDa ladder (MagicMark Protein Standard, Life Technologies). Ladder lanes not shown.

Transient expression of gM and gN in plasmid transfected GPL fibroblast cells demonstrated a co-localization of both proteins in the cytoplasm (Fig 3). Co-localization of gN with gMGFP was similar regardless of tag used for full length gN. Additionally, the N-terminal truncated gN, gN(s), also co-localized with gM in a similar manner (Fig 3I–3L). Importantly, this results is also observed in HCMV [61]. Immunoprecipitation (IP) studies of transiently expressed proteins confirmed specific protein:protein interaction between gM (GFP tag) and gN (mCherry or FLAG tagged). Immunoprecipitation assays were carried out with a commercial IP system, GFP trap (ChromoTek), which consists of coupled recombinant single domain anti-GFP antibody fragments derived from alpaca as monovalent matrices (agarose beads). In addition to normal antibodies, *Camelidae* make a second antibody called heavy chain antibody that lack a light chain and bind their antigen via a single variable domain. This approach reduces potential background associated with the use of regular antibodies in IP assays. Manufacturer’s protocol (ChromoTek) was followed to immunoprecipitate any proteins that interacted with gMGFP (Fig 4) or control GP84GFP [51]. Proteins were detected at approximately 50 kDa and 100 kDa that corresponded to gMGFP (Fig 4i, 4iii and 4iv). Both mCherry and FLAG tagged full length gN proteins were precipitated by interaction with gMGFP and both high molecular weight and lower weight species of gN were equally precipitated (Fig 4i and 4iii). Immunoprecipitation of truncated gN(s) indicated both glycosylated and non-glycosylated versions of gN interact with gM (Fig 4iii and 4iv) and that the complex is not dependent upon the N-terminal domain of gN. The IP results confirmed the suggested co-localization/interaction data seen in Fig 3. Control IP of GFP tagged GP84 [51] with gN(f)FLAG failed to show any specific interaction, see Fig 4(ii), which demonstrated specificity of the assay. We concluded that in GPCMV a functional gM/gN homolog complex is formed. In HCMV, the gM/gN complex is considered an important antibody target. The immune response to GPCMV gM/gN homolog complex was evaluated by a newly established ELISA (see section on immune response to GPCMV glycoprotein complexes).

### GPCMV gH(GP75)/gL(GP115)/gO(GP74) studies

**Fig 5:**
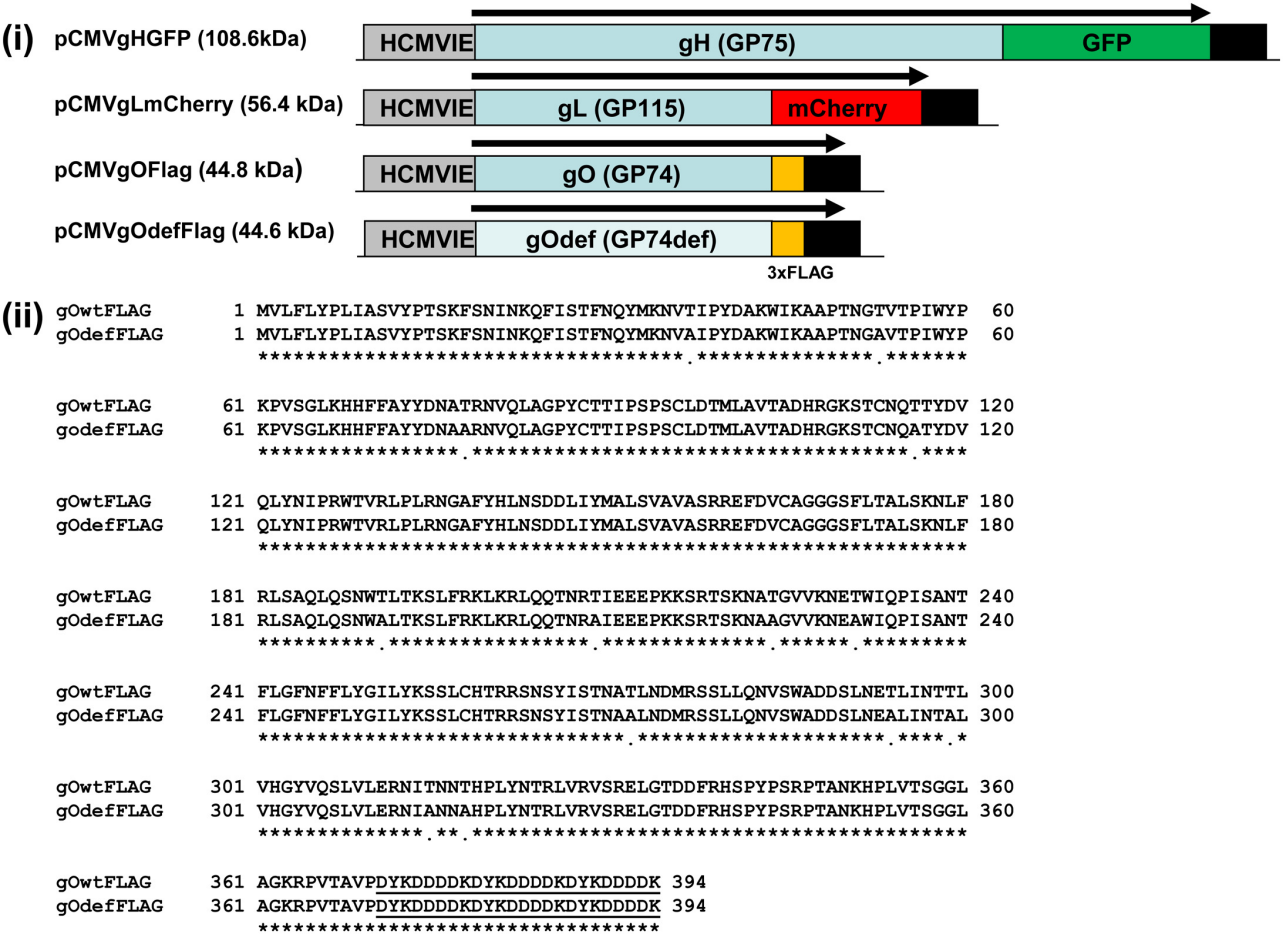
Transient expression constructs for GPCMV gH, gL, gO glycoproteins. **(i)** Shows the structure of the C-terminal tagged ORFs and expression plasmids: gH (GP75) was tagged with GFP; gL (GP115) was tagged with mCherry; gO (GP74) both wild type and mutant ORFs were tagged with 3xFLAG tag. **(ii)** BLAST alignment of wild type and mutant gO ORFs. The 13 predicted N-glycosylation sites were knocked out in the mutant (gOdef) by substitution of an alanine (A) in place of threonine (T) to disrupt the N-glycosylation recognition sequence (N-X-T/S).

In HCMV, the gH glycoprotein interacts with gL and gO to form the gCIII triplex complex or with gL, UL128, UL130 and UL131 proteins to form the pentameric complex [32, 62, 63]. While both complexes are important immune targets, our focus in this paper is on the GPCMV homolog gH/gL/gO complex. Expression plasmids (Fig 5) or recombinant defective adenoviruses encoding GPCMV gH (AdgHGFP, GP75 GFP C-terminal tag) and gL (AdgLmCherry, GP115 mCherry C-terminal tag) were used in conjunction with wild type or defective mutant gO (GP74 3xFLAG C-terminal tag) expression plasmids to determine interactions between the homolog proteins in the presence and absence of virus.

**Fig 6:**
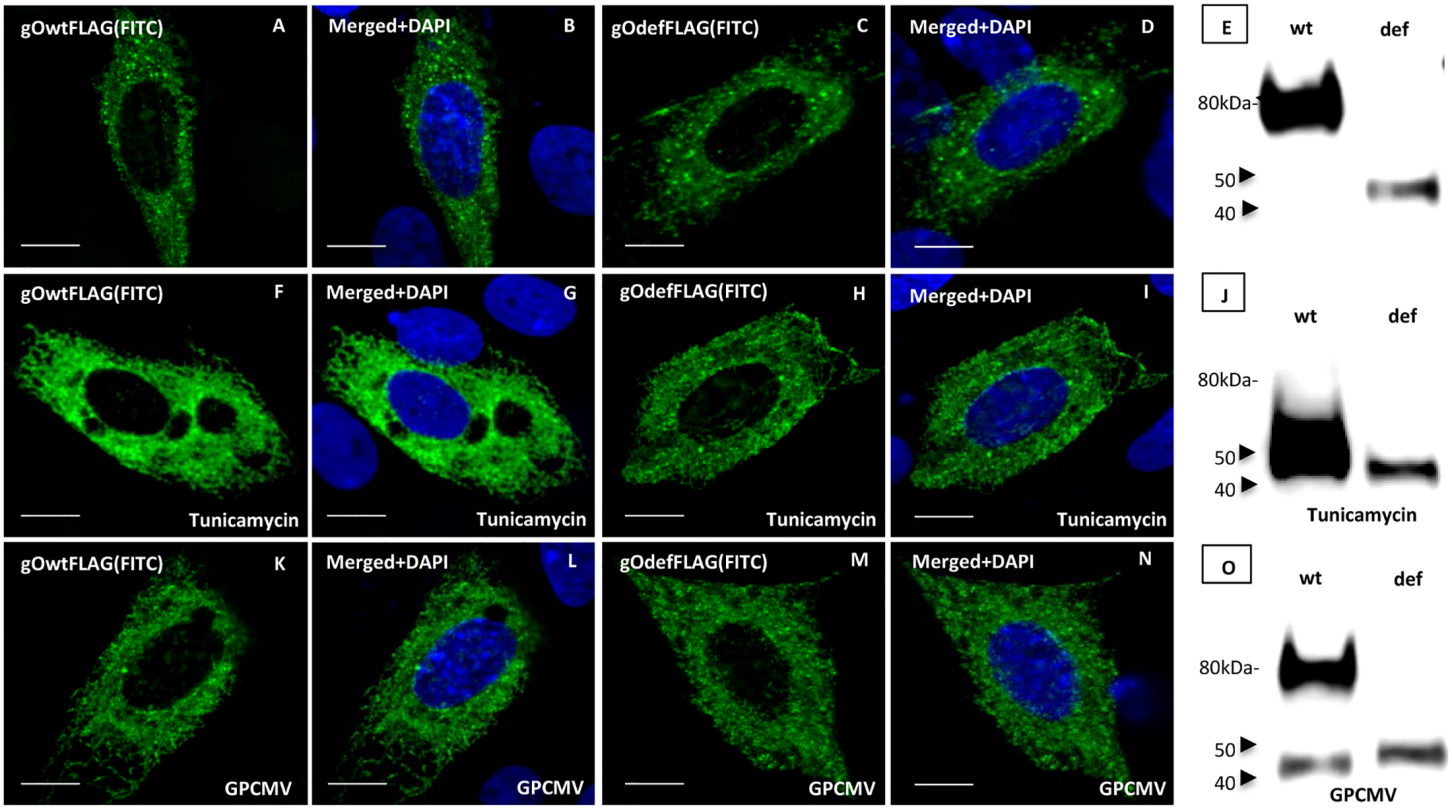
Transient expression of wild type or mutant gO in the presence or absence of GPCMV. The cellular location and molecular weight of gO protein was investigated by transient expression studies. Panels E, J and O are western blots for wild type or mutant gO using anti-FLAG antibody. Other panels are immunofluorescene images of wild type and mutant gO protein cellular localization by transient plasmid expression in GPL cells (A-D); GPL cells the presence of tunicamycin (glycosylation inhibitor, 2.5 ug/ml) (F-I); GPL cells plus GPCMV (K-N). Matched paired panels for gO (FITC) or gO (FITC) and DAPI (merged): A and B; C and D; F and G; H and I; K and L; M and N. Western blots: for wild type or gO mutant (E); wild type or gO mutant in the presence of tunicamycin (J); wild type or gO mutant in the presence of GPCMV (O).

As with HCMV, the GPCMV gO homolog protein, encoded by GP74, is predicted to be extensively modified post-translationally by N-linked glycosylation (codon recognition sequence NXT/S, 13 potential sites), see Table 1 and S2 Fig A defective gO ORF was generated synthetically which removed all 13 potential N-glycosylation sites by codon modification from T to A in the NXT motif. Fig 5(ii) shows BLAST alignment between wild type and mutant GP74 proteins. Both wild type and mutant gO were C-terminal FLAG tagged, underlined in Fig 5(ii). Separate transient expression studies of wild type and defective gO were performed under various conditions (Fig 6). Both versions of the gO protein have similar cellular localization (Fig 6). Western blot assays of transiently expressed gO proteins indicated that the wild type protein has a higher molecular weight (80kDa) than the mutant gO (45 kDa), but the predicted difference in size between the two proteins was 0.2 kDa. Additionally, both proteins should be approximately 45 kDa in size (Fig 6). Potentially, post-translation N-glycosylation associated with wild type, but not the mutant gO, (Fig 6E) accounted for the difference in size. When glycosylation inhibitor tunicamycin was included in experiments, the majority of the wild type gO protein was the lower molecular weight protein species (45 kDa). The mutant gO molecular weight size was unchanged (Fig 6J) which suggested that the difference in molecular weight was due to N-glycosylation. Transient expression of gO (wild type and defective) in the presence of virus resulted in the ability to detect both versions of wild type gO (N-glycosylated and non N-glycosylated), whereas the defective gO remained unchanged (Fig 6O). We concluded that gO is N-glycosylated and during GPCMV infection the level of gO N-glycosylation is modified possibly by disruption or modification of the secretory pathways but this awaits further investigation.

**Fig 7:**
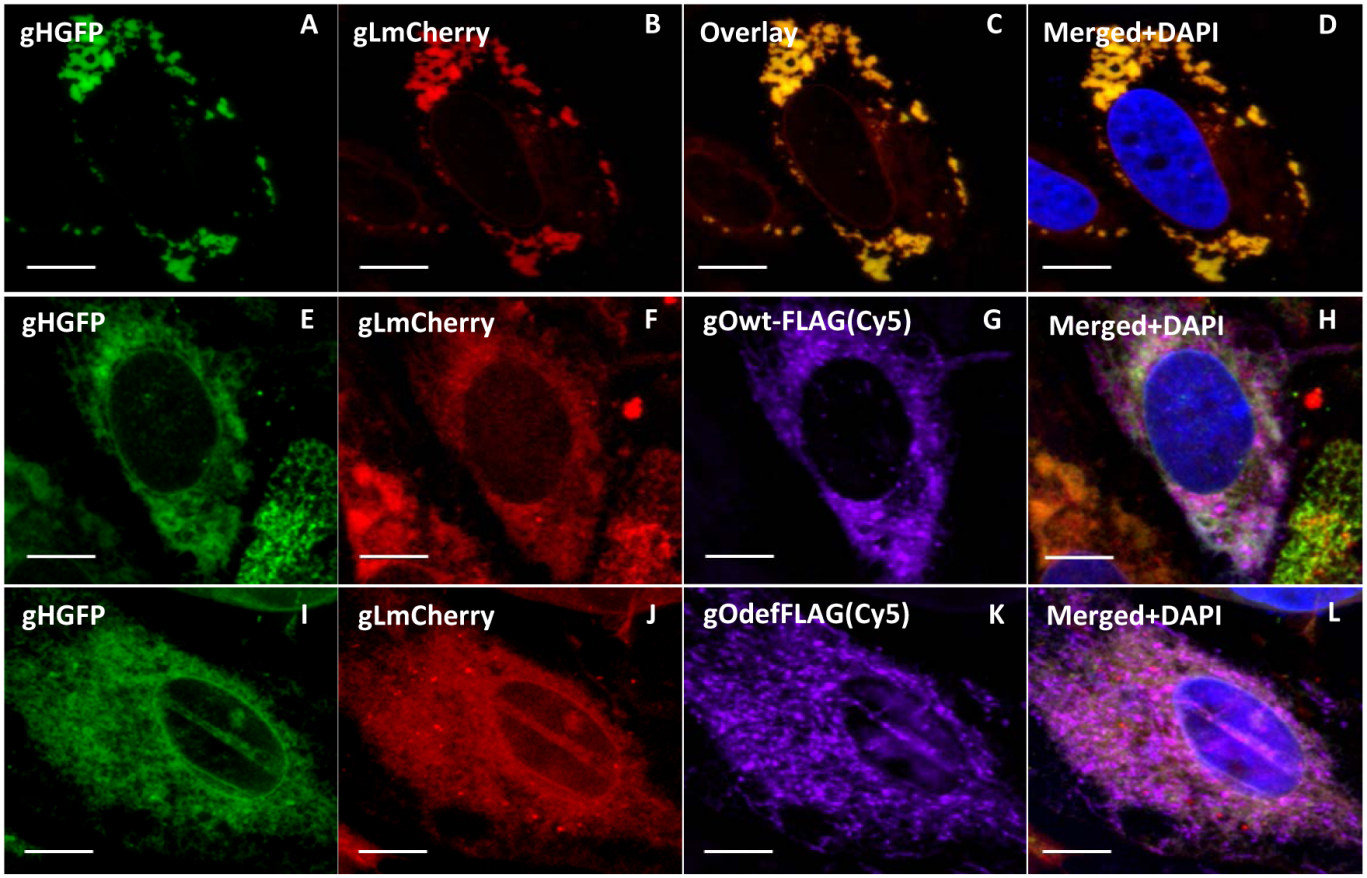
Transient expression and cellular co-localization of GPCMV gH, gL and gO. Expression plasmids described in Fig 5 were used to transiently express the viral glycoproteins in GPL cells. Panels **A-D**, gHGFP and gLmCherry co-expression. A and B cellular location of gH and gL within the same cell respectively. **C**, overlay of A and B. **D**, merge of overlay and DAPI stain to indicate location of nucleus. Panels **E-H**, gHGFP, gLmCherry and gOFLAG. Individual cellular localization panels **E, F** and **G**. Merged panel **H**, shows co-localization of all three protein and DAPI stained nucleus. Panels **I-L** are for gHGFP, gLmCherry and gO(def)FLAG. Individual panels **I**, **J** and **K**. Merged image (**L**) for all three panels plus DAPI stained nucleus.

**Fig 8:**
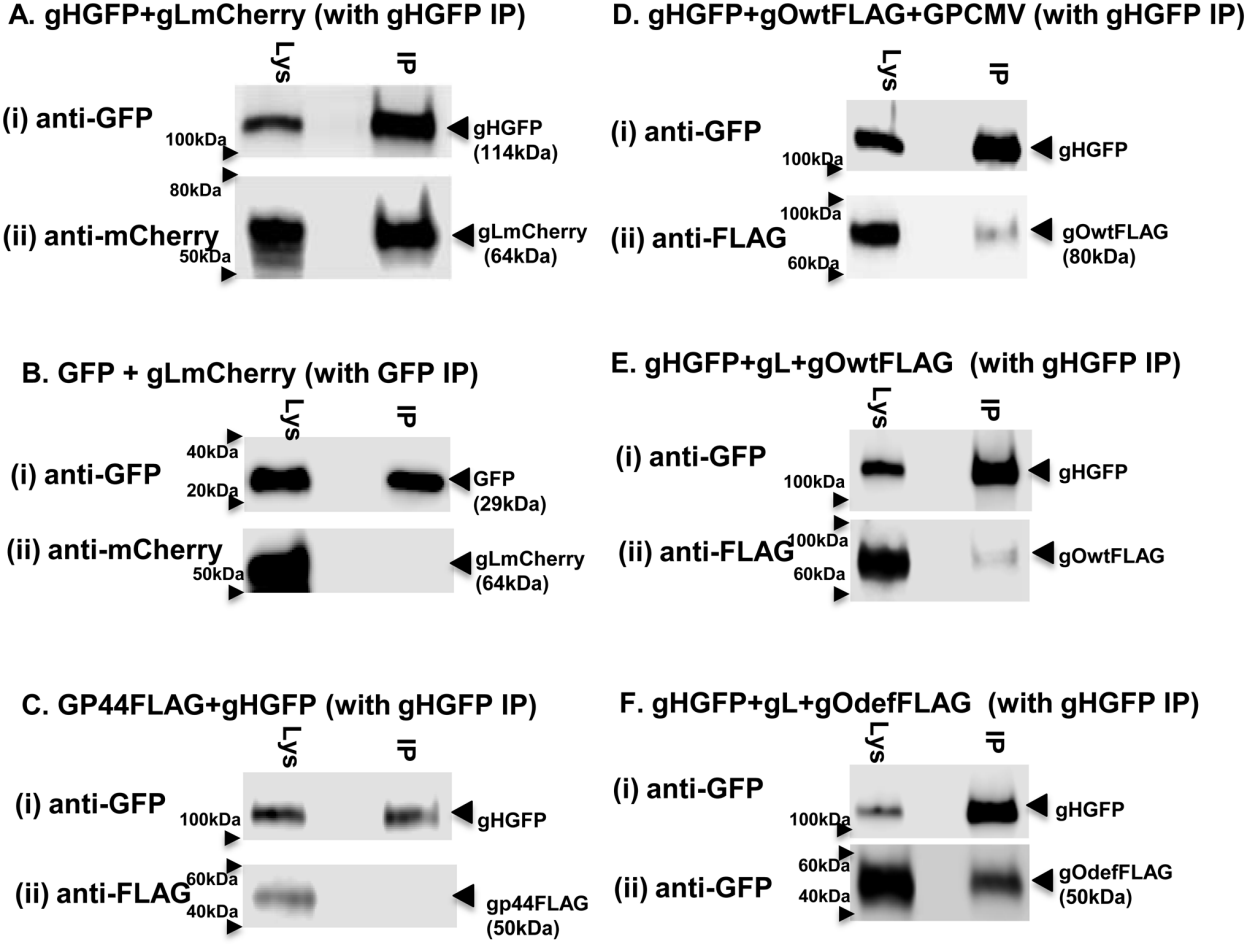
GPCMV gH/gL/gO complex formation and immunoprecipitation (IP) assays. All immunoprecipitations were performed with GFP-Trap (ChromoTek) as described in materials and methods to demonstrate protein:protein interaction with GFP tagged gH. Proteins were detected by specific antibodies to tags (GFP, mCherry or FLAG). Transient expression assays on GPL cells were carried out in different plasmid combinations as indicated A-F. Samples shown for each combination are total cell lysate (Lys) and IP from total cell lysate. Control proteins were GFP or FLAG tagged GP44 (polymerase subunit protein). **A**. gHGFP and gLmCherry IP: **A(i)** gHGFP western; A(ii) gL(mCherry) western. **B**. GFP control and gLmCherry control: B(i) GFPwestern; B(ii) gL (mCherry) western. **C**. GP44 and gHGFP: (i) gHGFPwestern; (ii) GP44(FLAG) western. **D**. gHGFP and gO (wt) in the presence of GPCMV: (i) gH western; (ii) gO(FLAG) western. **E**. gHGFP, gLmCherry and gO (wt): (i) gH western; (ii) gO (FLAG) western IP. **F**. gHGFP, gLmCherry and gO(def): (i) gH western; (ii) gO(def) western.

Fluorescence and immunofluorescence assays of transiently expressed GPCMV gH, gL and gO homolog proteins demonstrated that gH and gL co-localized in the cytoplasm of GPL fibroblast cells (Fig 7A–7D). Consequently, initial interactions between gH/gL would not appear dependent upon gO. However, in the absence of gO, gH/gL appeared to form aggregates in the cytoplasm. In the presence of gO, the gH/gL proteins are more uniformly distributed in the cytoplasm (Fig 7E–7H). Co-localization of gO with gH/gL was independent of the N-linked glycosylation state of gO as both wild type and mutant gO co-localized with gH/gL in a similar manner (Fig 7I–7L). In order to further evaluate specific protein:protein interactions, immunoprecipitation studies of transiently expressed proteins (gH, gL and gO) were carried in GPL cells using GFP trap (ChromoTek) to pulldown all proteins that interacted with GFP tagged gH or GFP control. Initial studies confirmed specific protein:protein interaction between gH (GFP tag) and gL (mCherry tag) (Fig 8A), whereas control IP of GFP and gL failed to pulldown gL (Fig 8B). Pulldown studies also demonstrated a specific interaction of gO with gH and gL (Fig 8D and 8E) which was not dependent upon the presence of virus (Fig 8E). As expected, based on co-localization studies, the gO mutant protein (GP74def) was also immunoprecipitated which demonstrated complex formation was independent of N-glycosylation status (Fig 8F). An additional control IP assay with FLAG tagged GP44, viral polymerase subunit protein [51], and gHGFP demonstrated that there was no non-specific interactions with gH (Fig 8).

**Fig 9:**
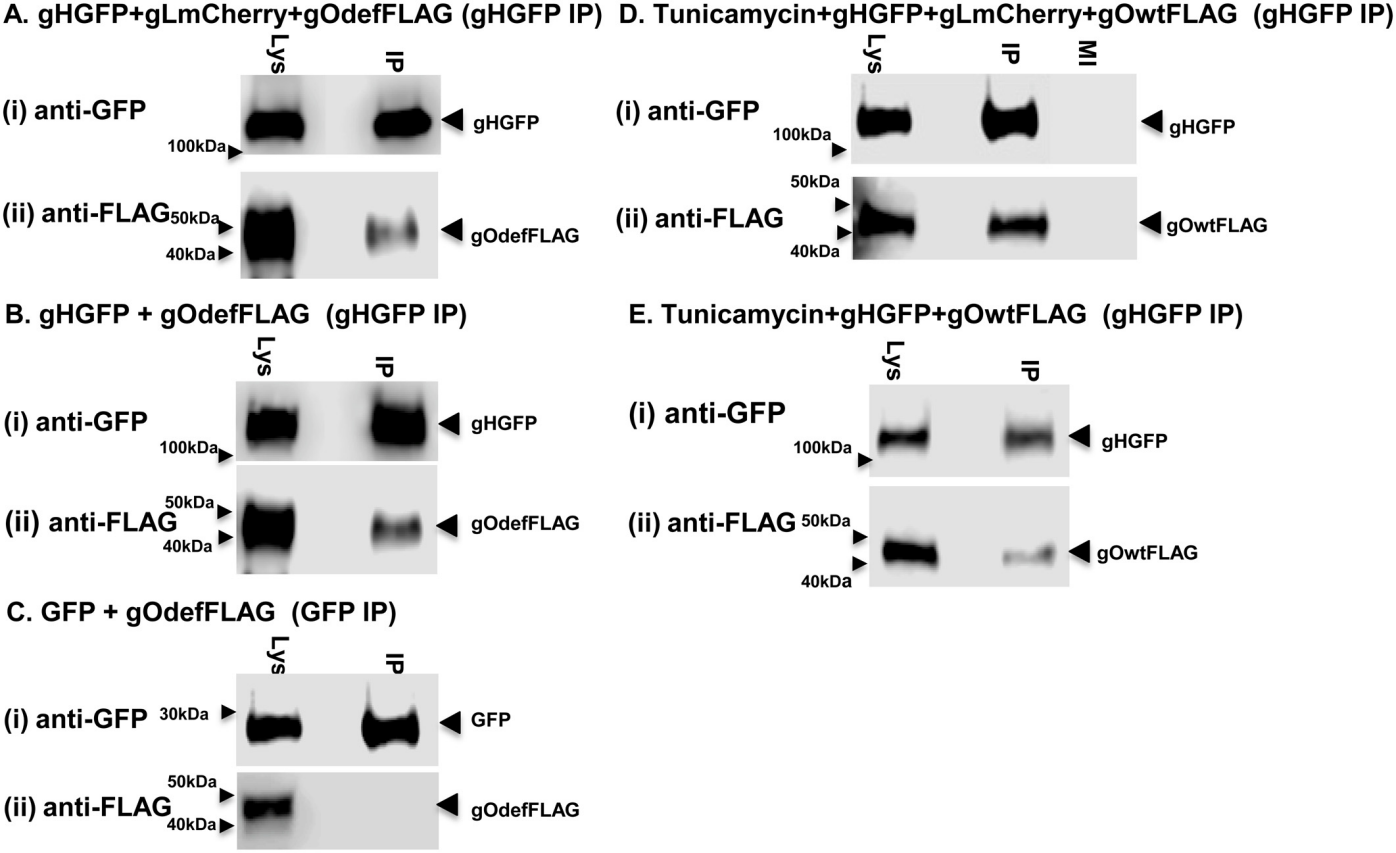
GPCMV gH/gO complex formation and immunoprecipitation (IP) assays. All immunoprecipitations were performed with GFP-Trap (ChomoTek) as described in materials and methods. Proteins detected by specific antibodies to tags (GFP or FLAG). Transient expression assays were carried out in different plasmid combinations as indicated A-E. Samples shown for each combination are total cell lysate (Lys) and IP from total cell lysate. Control protein was GFP. gHGFP and gOdefFLAG IP in presence (**A**) or absence (**B**) of gLmCherry: A(i) gH western and A(ii) gO (def) western; B(i) gHwestern; (ii) gO (def) western. **C. GFP and** gO(def) FLAG control: C(i) GFPwestern; C(ii) gO(def) western. **D**. gHGFP and gO(wt) FLAG in the presence of gLmCherry and tunicamycin: D(i) gH western; D(ii) gOgO western. **E**. gHGFP and gO(wt) FLAG in presence of tunicamycin: E(i) gH western; E(ii) gOwestern.

A series of further transient co-expression studies were carried out in GPL cells with gHGFP and gO (both wild type and mutant versions) to evaluate the ability of gH to complex with gO in the absence of gL. Fig 9 demonstrated that the gO mutant formed a dimer complex with gH. However, in order for wild type gO to form a detectable complex with gH, the glycosylation inhibitor tunicamycin needed to be present during transient expression. If wild type gO formed a dimer complex with gH in the absence of tunicamycin then it was below the sensitivity of detection for our IP assay (data not shown). We concluded that a homolog gH/gL complex can form in GPCMV and that a triplex with gO is possible but is independent of the N-glycosylation state of gO. Additionally, gH can form a dimer with gO but only with non-N-glycosylated gO but the significance of this novel dimer awaits further investigation, as does the influence of gH glycosylation status upon protein interaction. A specific gH/gO dimer complex has not been identified in HCMV and potentially this might be due to the lack of studies with a mutant gO protein deficient in predicted N-glycosylation sites. This awaits studies in HCMV with specific gO mutants. The immune response to GPCMV gH/gL homolog complex is potentially an important component of the protective immune response in convalescent animals. The gH/gL immune response was evaluated by a newly established ELISA (see section below on immune response to GPCMV glycoprotein complexes).

### Immune response to GPCMV glycoprotein complexes

**Fig 10:**
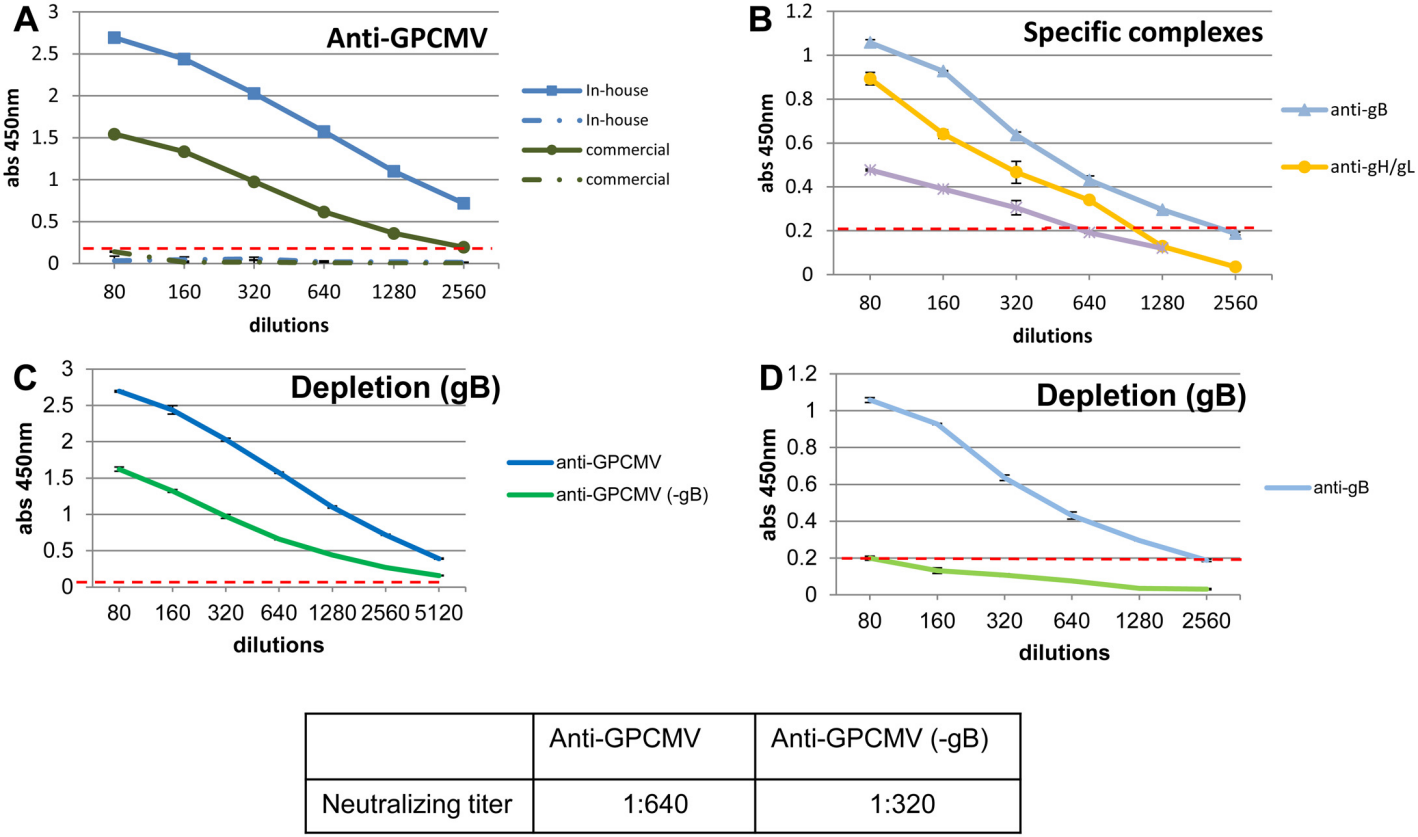
Antibody immune response of convalescent guinea pigs to GPCMV and GPCMV glycoprotein complexes determined by ELISAs. Pooled sera from GPCMV infected convalescent animals was used to evaluate immune response to GPCMV **(A)** or to specific glycoprotein complexes **(B)**. **(A)** Anti-GPCMV ELISA. The immune response to GPCMV antigens was analyzed by an in-house ELISA (blue line square) compared to commercially available GPCMV ELISA kit from Bioexpress (green line circle). GPCMV sera negative for both assays are shown in blue and green dotted lines. **(B)** Immune response of convalescent pooled guinea pig serum to individual glycoprotein complexes. Anti-gB, light blue line (triangle); Anti-gH/gL, orange line (closed circle); Anti-gM/gN, purple line (star). Convalescent sera depleted of anti-gB antibody (-gB) (as described in materials and methods) was retested by ELISA to **(C)** anti-GPCMV ELISA (solid green line) to demonstrate retention of antibody response to other viral antigens, and **(D)** anti-gB specific ELISA (solid light green line) to demonstrate gB depletion compared to the original undepleted sera. All sera were diluted from 1:80 to 1:2560 in doubling dilutions. Control sera from animals negative for GPCMV were used for base line in all assays. ELISAs performed as described in materials and methods. Base level for background indicated by horizontal dotted red line.

The immune response to GPCMV and viral glycoprotein homolog complexes were investigated for pooled sera from animals convalescent for GPCMV. An in-house anti-GPCMV ELISA (as described in materials and methods) showed high sensitivity for viral antigens when compared to a commercial anti-GPCMV ELISA kit (Xpress Bio), see Fig 10A. The in-house anti-GPCMV ELISA sensitivity level (end point >1:2560) was twice as great as the commercial ELISA kit (end point 1:1280), based on net OD values and the negative cut-off of 0.2. The OD values of negative serum were similar for both in-house and commercial assays. Neutralizing antibody assay of GPCMV convalescent serum on GPL cells demonstrated a neutralizing titer of 1:640 (Fig 10). This was similar to values previously reported [46]. The immune response to GPCMV gB, gH/gL and gM/gN homolog complexes was evaluated by newly established ELISAs as described in materials and methods (Fig 10B). Convalescent sera from GPCMV infected animals demonstrated a specific immune response to GPCMV viral glycoprotein complexes. The GPCMV gB specific ELISA had an endpoint of 1:1280 dilution. The gB protein is the immunodominant glycoprotein complex in both HCMV and GPCMV [64, 65] and our assays confirmed this observation. Depletion of anti-gB antibodies from convalescent sera was confirmed by gB specific ELISA (Fig 10D). Additionally, S7 Fig demonstrated by Western blot the successful depletion of the gB specific immune response. However, the gB antibody depleted sera in anti-GPCMV ELISA only showed a slight decrease in endpoint titer (Fig 10C). Furthermore, depleted sera retained neutralizing capability (1:320) which emphasized the importance of other glycoprotein target antigens in protection against GPCMV infection. In HCMV, the immune response is directed to the gH/gL complex rather than the gH protein alone [56,57]. A GPCMV gH/gL ELISA demonstrated a specific immune response with an endpoint of 1:640 serum dilution (Fig 10B). A relatively robust immune response is similar to that seen in HCMV convalescent sera. Although gH/gL can complex with gO to form a triplex, we found that gO is not required as a component in the ELISA assay (data not shown). It remains to be determined if the triplex is required in GPCMV to enhance the immune response. In preliminary immunogenicity studies it would appear that gH/gL is immunogenic in the absence of gO (data not shown; Choi and McGregor unpublished observation). An ELISA to gH only could not give an accurate reading above background at the dilutions used. However, specific immune response to gH could be demonstrated by western blot analysis (data not shown). The immune response to the gM/gN complex was lower than the response to gB or gH/gL with an end titer of 1:320 (Fig 10B). Anti-gB and anti-GPCMV antibodies have been demonstrated to neutralize virus infection on fibroblast cells. Here we demonstrate specific immune response to the various glycoprotein complexes as well as neutralizing antibodies that target viral glycoproteins in addition to gB. The importance of these specific neutralizing antibodies in the context of virus entry into various cell types awaits further study.

### Characterization of the essential nature of GPCMV glycoproteins

In order to determine the essential nature of the GPCMV glycoproteins, each gene was individually knocked out by targeted mutagenesis of the GPCMV BAC in bacteria using shuttle vectors carrying a kanamycin (Km) drug resistance marker to disrupt each ORF as described in materials and methods. The specific site of disruption for each glycoprotein is summarized in Table 1. Targeted recombination knockout of each glycoprotein gene on the GPCMV genome was performed in separate transformation reactions in both first generation and second generation GPCMV BACs and gene knockouts were selected by insertion of the Km cassette into the GPCMV BAC genome. S1 Fig shows the location of the genes in the viral genome as well as the restriction enzyme profile analysis of the various mutant GPCMV BAC clones compared to wild type genomes. Analysis shown is for the mutagenesis of the second generation GPCMV BAC [21]. Modifications to the BAC genomes for each mutant were as expected. More in depth analysis of the characterization of the GPCMV BAC profiles is provided in the Materials and Methods section. The modified loci for the GPCMV glycoprotein gene mutants were also confirmed by specific PCR analysis of wild type and disrupted glycoprotein genes using common flanking primers for each gene. S3 Fig shows the predicted sizes of PCR products for both wild type and mutated glycoprotein genes using common flanking primer pairs described in S1 Table. S4 Fig shows the actual PCR results for the GPCMV glycoprotein BAC wild type and mutant loci using the common flanking PCR primer pair for each gene. The actual size modification to the PCR product for each knockout glycoprotein locus (*GP55*, *GP73*, *GP74*, *GP75*, *GP100* and *GP115*) in comparison to the wild type locus was as predicted. The GPCMV mutant PCR products were additionally sequenced to further verify knockout mutations (data not shown).

**Fig 11:**
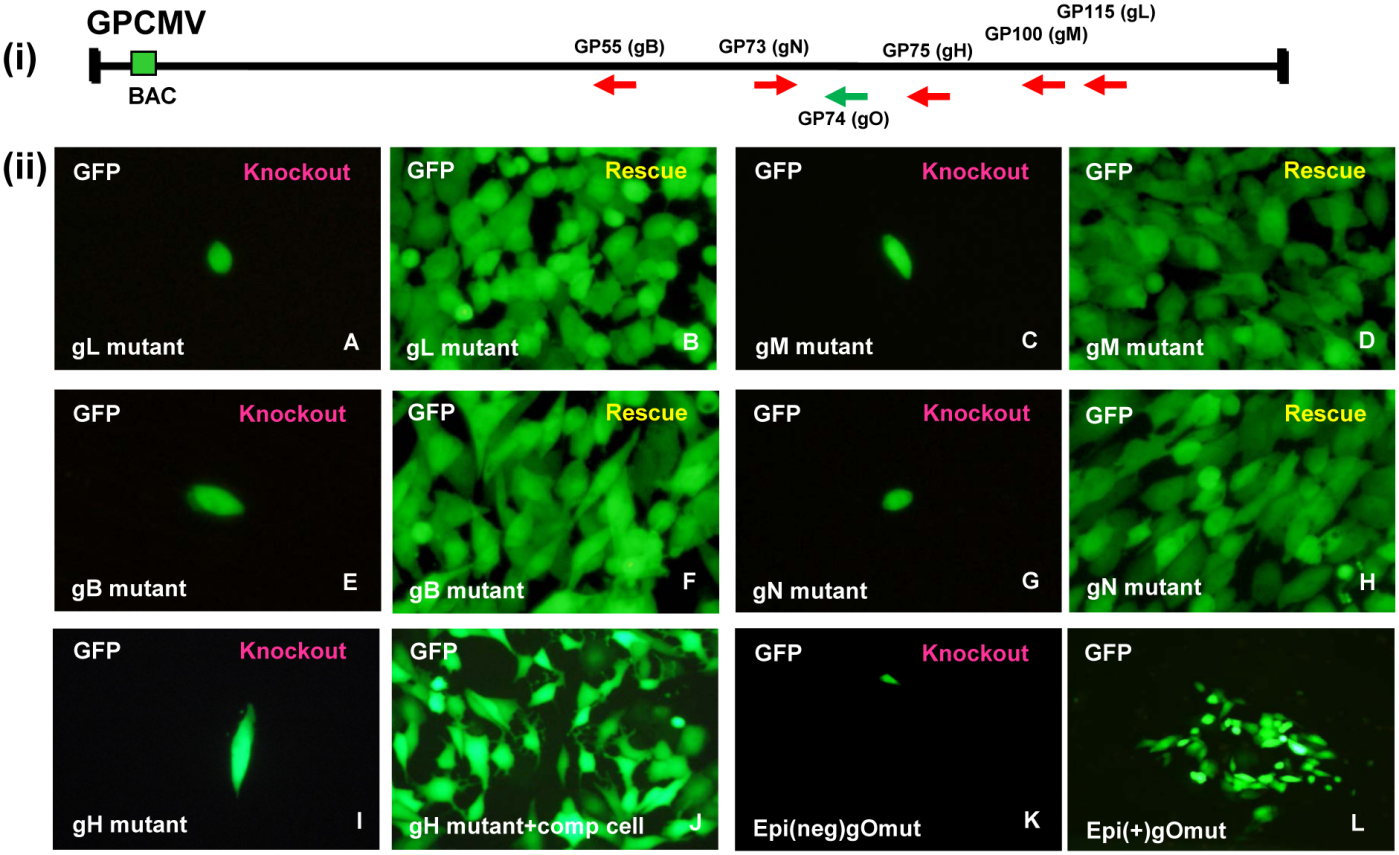
Transfection of glycoprotein mutant KO GPCMV BACs onto GPL cells. Individual mutant GPCMV BACs were separately transfected onto GPL fibroblast cells to regenerate virus. GFP reporter gene encoded in the viral genome enabled real time tracking of the development of virus from individual transfected cells. Glycoprotein mutant GPCMV BACs were either transfected individually (panels A, C, E, G, I, K and L) or in combination with a rescue plasmid encoding a wild type locus to restore the mutant back to wild type phenotype where GFP virus could be detected spreading across the cell monolayer (panels B, D, F and H). The gH mutant was also transfected onto a cell line expressing gH *in trans* to support virus growth (panel J). A gH rescue virus was also generated by co-transfection with a rescue locus plasmid (data not shown). Panels K and L show the outcome for a gO knockout mutant based on the back drop of a virus carrying (L) or lacking (K) epithelial cell tropism. Only gO mutant GPCMV with epithelial tropism could grow on GPL cells. A rescue virus of panel K was generated by co-transfection of the gO GPCMV mutant with a wild type locus plasmid to restore wild type virus phenotype (data not shown). Images taken between day 16–18 post transfection.

DNA from each individual knockout mutant GPCMV BAC was transfected separately onto GPL cells to determine if the specific glycoprotein gene knockout was lethal for the virus. Duplicate clones were generated for each mutant (data not shown). Additionally, each mutant BAC was co-transfected with the appropriate rescue plasmid or rescue PCR product encoding the respective wild type glycoprotein gene to generate rescue virus from lethal knockout mutants (see materials and methods). The results shown in Fig 11 demonstrated that all of the encoded GPCMV glycoproteins (gB, gH, gL, gM, gN, gO) are essential for virus replication in tissue culture. The presence of a GFP reporter gene encoded in the viral genome enabled real time tracking of virus development and spread across the cell monolayer. Non-infectious GPCMV BAC mutants remained as a single GFP positive transfected cell during the course of the assay. Rescue of the mutant locus enabled development of rescue virus which was detected by GFP virus spread (Fig 11). Overall, the results for GPCMV are similar to that obtained for HCMV [27, 66] with the exception of gO which is essential in GPCMV lab adapted virus (virus lacking epithelial tropism with defective UL128-131 homolog locus) but non-essential/ semi-essential in virus with epithelial tropism (intact homolog *UL128-131* genes). All knockout GPCMV mutants could be successfully rescued by co-transfection of the appropriate rescue plasmid to generate wild type virus (Fig 11).

The viable gO mutant virus was highly cell associated and produced virtually no detectable cell release virus. Viral spread on GPL cells (and primary guinea pig embryo fibroblasts, data not shown) was relatively restricted to primary plaques and was incapable of spreading across the full monolayer of cells. This was presumably due to the limited ability of the fibroblast cells to enable virus entry via endocytosis pathway. In epithelial cells, the gO mutant virus could successfully spread across the complete monolayer but had delayed growth kinetics compared to wild type virus (data not shown, Coleman et al., paper in preparation). Given the lack of cell release virus produced by the gO mutant virus, it is likely that the gO protein has a role in viral maturation but confirmation awaits further study.

## Discussion

This is the first report of a systematic knockout of the encoded glycoprotein genes of an animal cytomegalovirus and characterization of their glycoprotein complexes. The level of identity that exists between HCMV and GPCMV glycoproteins, as well as the conserved essential nature of viral proteins, implies that both HCMV and GPCMV glycoproteins have similar function in the viral life cycle. Since homolog glycoproteins to gB, gH, gL, gO, gM and gN are encoded by other animal CMV, it is probable that homolog glycoproteins from other species have a similarly conserved essential nature. In HCMV, variations in specific glycoprotein amino acid sequences have been identified in different clinical isolates of HCMV vs lab strains of HCMV. Two glycoproteins in particular (gN and gO) exhibit the greatest variation in sequence [67]. A recent analysis of the complete genome sequence of plaque purified MCMV (Smith) passaged in tissue culture, sequenced, passaged in mice and then sequenced directly from salivary gland source did not reveal any changes in the glycoprotein gene sequences [68]. An analysis of GPCMV gN and gO genes from ATCC virus in comparison to salivary gland derived GPCMV (serially passaged in guinea pigs over 12 passes and maintained in guinea pigs for over 20 years) did not demonstrate any variation from the GPCMV ATCC sequence of these predicted proteins [69] (McGregor, unpublished data). This suggests that GPCMV glycoprotein gene sequences are relatively stable within the same strain as is the case for MCMV. A new strain of GPCMV (CIDMTR strain) has recently been identified [70] and shows the greatest changes in codon sequence in the gO ORF compared to GPCMV 22122 strain (303/374 codons, 81% identity). Analysis by BLAST alignment (data not shown) indicates that the majority of changes are in the N-terminal sequence of the first 60 amino acids. Importantly, these changes did not modify the conserved number of N-glycosylation sites in GPCMV gO. Therefore it is unlikely that the changes in the gO sequence between GPCMV strains are likely to impact on the basic functional nature of the protein. Potentially, specific changes in the gO sequence could enable a modified interaction with gH/gL and subsequently enable gH/gL to better associate with components of the homolog pentameric complex (GP129, GP131 and GP133 proteins) as recently proposed for HCMV [71]. Based on our studies, a modified N-glycosylation status of gO could potentially influence the ability of gO to interact with gH which might also have a role in enabling gH/gL to interact with components of the homolog penatmeric complex. In GPCMV infected cells, both glycosylated and non-glycosylated versions of gO were detected in equal abundance. In contrast, transient expression studies of wild type gO only detected the N-glycosylated version of the protein. The significance of the N-glycosylated and non-N-glycosylated gO protein in CMV infection awaits further study in both GPCMV and HCMV. In clinical HCMV strains, studies by Johnson and colleagues suggest that the gO protein is not necessarily a component of the viral particle but is required as a chaperone protein to efficiently get gH/gL incorporated into the virion [72]. Studies by Zhou et al. [73] indicate that the isoform of gO in different strains of HCMV dictate the ratio of gH/gL complexes in viral envelope (gH/gL/gO vs gH/gL/UL128-131 pentameric). Potentially, there might be a variation in the ratio of homolog complexes between the two GPCMV strains. However, this awaits further study and will require the generation of a GPCMV gO specific antibody.

HCMV gO protein is extensively N-glycosylated with 18 potential of N-X-S/T sites [32] and this may provide gO protein with a more effective interaction with the endoplasmic reticulum (ER) calnexin chaperone protein system [74]. This in turn may enhance the movement of gO or gH/gL/gO complex through the ER resulting in more efficient virion maturation and egress. In this context, it is perhaps unsurprising that the modification of the predicted GPCMV gO to remove the 13 N-linked glycosylation sites did not alter the ability of gO to interact with gH and gL homologs. Generation of a recombinant virus encoding N-glycosylation deficient gO may provide further insight into the role of N-glycosylation to the viral life cycle and specifically viral maturation. It is interesting to note that a GPCMV gO (GP74) knockout mutant either completely abolished the ability to make virus (lab adapted GPCMV) or impaired the ability to generate detectable cell released virus (epithelial tropic GPCMV). This sharply contrasts with HCMV, where there is no lethal phenotype associated with a gO knockout in lab adapted HCMV [27]. However, in clinical HCMV strains, a gO knockout is more impaired for growth [75]

In our GPCMV ELISA assays, we demonstrated that the antibody immune response in convalescent animals is directed to various glycoprotein complexes (Fig 10). In HCMV infection, the gB protein is the immunodominant target antigen and an ELISA to the full length GPCMV gB (Fig 10B) would suggest that this is also the case for GPCMV. Anti-GPCMV gB specific antibodies, either monoclonal or polyclonal have been shown to have specific neutralizing activity [42, 44]. However, vaccine strategies based solely on GPCMV gB fail to fully protect against congenital infection despite inducing high neutralizing antibody titer [42, 43, 54]. Consequently, there is a need for further development of strategies to other neutralizing target antigens.

In HCMV, both gH/gL and gB complexes are neutralizing antibody targets. Recently, the GPCMV gH/gL complex has also been shown to generate neutralizing antibodies in mice [76] which would potentially imply that guinea pigs also generate neutralizing antibodies to gH/gL but this awaits further verification using guinea pig gH/gL specific antisera lacking antibodies to other viral proteins. In convalescent GPCMV infected animals, the GPCMV neutralizing titer for sera ranges between 1/160 to 1/640 (Choi and McGregor, unpublished data) but this contains antibodies to various viral proteins/ glycoproteins. The dissection of the neutralizing immune response to individual viral protein complexes requires serum depletion of antibodies to specific viral proteins as well as an evaluation of the specific immune response to individual complexes. Serum depletion of anti-gB antibodies demonstrated a decrease in overall titer in anti-GPCMV ELISA while presence of gB fell to undetectable levels in anti-gB specific ELISA. The neutralization titer of anti-gB depleted serum decreased 2 fold from 1:640 to 1:320 when compared to non-depleted serum. A similar 50% reduction in HCMV neutralizing titer on fibroblast cells is observed in gB antibody in human sera [77]. However, in guinea pig sera studies, the result suggests the importance of other glycoproteins such as gH/gL or gM/gN in protection against CMV. The availability of recombinant Ad vectors encoding gH and gL would make investigation of neutralizing antibodies to gH/gL a feasible next step for gH/gL complex studies. The importance of additional components of the homolog pentameric complex in enhancing immunogenicity/ neutralizing response on epithelial cells and prevention of congenital infection [38] is an important concern and the GPCMV pentameric complex is the subject of a related paper from our laboratory (Coleman et al, in preparation). Auerbach et al., [76] suggest that antibodies to the GPCMV gH/gL complex are sufficient to prevent congenital infection. However, published data from earlier studies that carry pregnancy to term (unlike studies by Auerbach and colleagues [76]) suggest that this is not the case. Unfortunately, the specific level of immune response to gH/gL was undefined in these earlier studies. Importantly, despite an antibody response to viral glycoprotein complexes (gB, gM/gN, gH/gL), prevention of congenital infection could not be fully attained in this model where pregnancy is taken to term [78] Unusually, Auerbach and colleagues [76] adopted an alternative strategy of a truncated in utero period of infection for their congenital GPCMV studies, without taking the animals to term. This potentially weakens their model and may account for the apparent discrepancy in protection rate against congenital infection compared to previously published data.

The gM/gN complex in HCMV is important for virus entry and the essential nature of these proteins in GPCMV would also suggest a similarly important role. HCMV and GPCMV gM and gN proteins exhibit the highest identity of all the homolog proteins as well as conserved predicted multiple transmembrane domains which would suggest conservation of structure and function. The lack of specific antisera to GPCMV gM/gN precludes the direct demonstration of this protein complex role in cell entry. However, the GPCMV gM protein does have heparin binding capability (data not shown) which is a conserved feature of other gM proteins [79]. In marked contrast to HCMV, it is interesting to note that the GPCMV gN predicted sequence does not vary between strain isolates (GPCMV 22122 vs CIDMTR) [70, 80]. As with HCMV, the GPCMV gN protein is post translationally modified with a number of predicted glycosylation sites (Table 1) and modification of the N-terminus leader sequence can impact of the post translational modification but not on the ability of gN to interact with gM. The significance of glycosylation on various GPCMV glycoproteins awaits further study but surprisingly does not impact on the ability to form complexes in transient expression assays. The GPCMV gN protein is slightly shorter than HCMV gN (133 vs 138 amino acids) but is also heavily glycosylated. It is of note that glycosylation of gN was initially thought to be restricted to HCMV and great ape CMV as the gN homolog is not glycosylated in Rhesus CMV or MCMV [81, 82]. Generation of recombinant HCMV encoding N-terminus truncated gN resulted in viable virus with non-glycosylated gN and a greater susceptibility to neutralizing antibodies which would suggest a critical role for gN in protecting against neutralizing antibodies [61]. Since GPCMV gN is N- and O-glycosylated, the role of gN and glycosylation in immune evasion could be investigated in future guinea pig model studies with recombinant viruses encoding wild type and mutant gN.

Overall, the results from these studies suggest that GPCMV forms glycoprotein complexes similar to HCMV and that there is conserved function and properties in these complexes between HCMV and GPCMV. The complexes are important immunogenic targets in guinea pigs convalescent for GPCMV and importantly the neutralizing immune response is not solely directed to gB. Knockout mutagenesis of the individual viral glycoproteins confirmed their essential nature. A second method of entry into cells potentially exists in GPCMV with intact tropism to epithelial cells which enables gO independent infection of both fibroblasts and other cell types. However, our current data could also suggest that the pentameric complex can inefficiently perform the same function as the triplex via the same mechanism. A pending paper from our lab on epithelial tropism mutants (Coleman et al.) would argue that the former is the case. Overall, these findings strengthen the guinea pig model for CMV and the continued development of intervention strategies based on this model.

## Conclusion

After more than fifty years of research, an effective intervention strategy against congenital cytomegalovirus remains an elusive goal. A complication with HCMV studies is the inability to directly utilize an animal model because of HCMV species specificity. GPCMV, a guinea pig specific virus, has emerged as highly relevant animal model because of the unique ability of the virus to cause congenital infection unlike mouse or rat models. An important aspect of CMV is the viral glycoproteins complexes that are present on the outside of the viral membrane. These are necessary for cell entry and are consequently important vaccine neutralizing target antigens. In this paper, we characterize the homolog glycoproteins (gB, gH, gL, gO, gM, gN) and respective complexes in GPCMV and demonstrate conserved function between GPCMV and HCMV glycoproteins. Based on newly established assays for GPCMV, the GPCMV glycoprotein complexes are highly immunogenic. Additionally, manipulation of the GPCMV genome was carried out via herpesvirus BAC technology to generate specific mutants to characterize essential function of individual glycoprotein gene which showed similarity to HCMV. Overall, these studies are an important step in the continued use of this model for development of vaccine intervention strategies against CMV.

## Supporting Information

S1 Fig Restriction profile analysis of wild type and glycoprotein gene knockout GPCMV BAC clones. Wild type GPCMV BAC was mutated as described in materials and methods to individually knockout each glycoprotein gene in separate GPCMV BAC clones. At least two independent mutants were analyzed per gene knockout but only one mutant is shown in the Fig (identical results were obtained for the second mutant, data not shown). Both *EcoR* I (Ec) and *Hind* III (Hd) restriction profile analysis were performed for each mutant GPCMV BAC but only one profile is shown for each mutant to reduce repetition. Specific band shift are indicated as original wild type band (yellow) and modified mutant band (red). (i) Map of the GPCMV genome with individual glycoprotein genes indicated: *GP55* (gB); *GP73* (gN); *GP74* (gO); *GP75* (gH); *GP100* (gM); *GP115* (gL). Red indicates the gene is essential and green that the gene is semi-essential/non-essential for viable virus (see results in Fig 11). Individual GPCMV gene mutant and wild type BAC comparative restriction enzyme digests (ii)—(vii). Specific modification made to each gene in the process of inserting a kanamycin gene marker is shown in S2 Fig and PCR analysis of the modified locus shown in S3 Fig.
(TIF)

S2 Fig Diagram showing modification to the individual glycoprotein genes to generate knockouts. A kanamycin cassette was PCR amplified with modified restriction sites and cloned into individual glycoprotein knockout shuttle vectors. In the case of gN (*GP73*), gO (*GP74*), gH (*GP75*), gM (*GP100*) and gL (*GP115*) the entire ORF was cloned and modified by kanamycin cassette insertion using indicated restriction sites. The GP75 ORF was modified by a collapse between two sites (*Nru* I and *EcoR* V). For the gB *(GP55)* the homolog AD-1 domain was PCR cloned as a shuttle vector with Km inserted into a unique *EcoR* V site as described in materials and methods to disrupt the ORF. The sizes of the original genes by PCR analysis are indicated and the sizes of the modified genes after kanamycin cassette insertion are also indicated (sizes verified by PCR in S4 Fig).
(TIF)

S3 Fig PCR analysis of GPCMV wild type and glycoprotein mutant gene loci. Common primers were used to amplify the genes of wild type and mutant GPCMV. PCR primers as described in materials and methods and S1 Table were used to verify that the individual glycoprotein genes had been correctly modified. PCR products of mutant and wild type genes were compared by agarose gel electrophoresis to verify specific modifications. Gels: (i) *GP74*, *GP75*, *GP100*, *GP115*. (ii) *GP73*. (iii) *GP55*. GPCMV BAC mutant and wild type GPCMV analysis via PCR. Sample lanes: (1) *GP74* wt; (2) *GP74* mutant; (3) *GP75* wt; (4) *GP75* mutant; (5) *GP100* wt; (6) *GP100* mutant; (7) *GP115* wt; (8) *GP115* mutant (9) *GP73* wt; (10) *GP73* mutant; (11) *GP55* wt; (12) *GP55* mutant.
(TIF)

S4 Fig Predicted glycoprotein signal peptide sequences. Various web based programs were used to predict the presence of a signal peptide sequence associated with individual proteins. (A) gB and (B) gH leader sequences determined by http://www.cbs.dtu.dk/services/SignalP/ [48]. (C) gM and (D) gL leader sequences determined by http://sigpep.services.came.sbg.ac.at/signalblast.html. (E) gN leader sequence determined by http://www.csbio.sjtu.edu.cn/bioinf/Signal-3L/ [49]. Data shown is the end result analysis from each program.
(TIF)

S5 Fig Use of chemical inhibitors to identify the specific class of transcript for genes GP100 (gM), GP74 (gO) and GP73 (gN). RT-PCR assays were carried out with GPCMV strain 22122 infected GPL cells in 6 well dish (moi = 1 pfu/cell) at different time points (6, 24 and 48 hr post infection) in the presence or absence of specific chemical inhibitors. Cycloheximide (CHX, 100 μg/ml) was used to prevent transcription of all but the IE transcripts and phosphonoacetic acid (PAA, 200 μg/ml) was used to prevent late transcripts as described in materials and methods. RT-PCR was carried out as described in materials and methods. Lanes: 1, bp ladder (Invitrogen); 2, mock infected; 3, 6 hour CHX treated; 4, 24 hour CHX treated; 5, 24 hour PAA treated; 6, 48 hour PAA treated; 7, no template control; 8, infected cell lysate no reverse transcriptase stage; 9, untreated (no inhibitor) GPCMV infected cell lysate. GP122 (IE2) RT-PCR is a positive control for GPCMV at specific assay time points treated with inhibitors. GAPDH is a positive cellular RNA control for all time point samples.
(TIF)

S6 Fig Comparison of the predicted transmembrane domains of gM and gN proteins in HCMV and GPCMV. The predicted amino acid sequences for HCMV and GPCMV gM and gN proteins were analyzed for potential transmembrane domains by the web based program TMHMM Server v. 2.0 Prediction of transmembrane helices in proteins (http://www.cbs.dtu.dk/services/TMHMM/). Potential transmembrane helices indicated in red in alignment with the predicted protein sequence (N to C terminal).
(TIF)

S7 Fig Western blot analysis of anti-gB depleted GPCMV convalescent sera. Anti-GPCMV sera depleted for anti-gB antibodies by preabsorption against Ad-gB transduced HEK 293 cells was verified for depletion by Western blot analysis as described in Materials and Methods. Lanes 1, 4, 7 mock infected GPL cells; Lanes 2, 5, 8 Ad-gB transduced GPL cell lysates (moi = 20 TDU/cell); Lanes 3, 6, 9 late stage GPCMV infected GPL cell lysates (moi = 1 pfu/cell). GPCMV convalescent sera (1:500) used for lanes 1–3, anti-gB depleted GPCMV sera (1:100) used for lanes 4–6. GPCMV gB monoclonal antibody (29–29) used for lanes 7–9 (1:500). Black arrow shows gB.
(TIF)

S1 Table Oligonucleotides used for GPCMV PCR and RT-PCR. (DOC)
