# Correction: Viral Glycoprotein Complex Formation, Essential Function and Immunogenicity in the Guinea Pig Model for Cytomegalovirus

**DOI:** 10.1371/journal.pone.0137942

**Published:** 2015-09-08

**Authors:** Stewart Coleman, Julia Hornig, Sarah Maddux, K. Yeon Choi, Alistair McGregor

There is an error in Fig 10. Please view the correct Fig 10 here.

**Fig 10:**
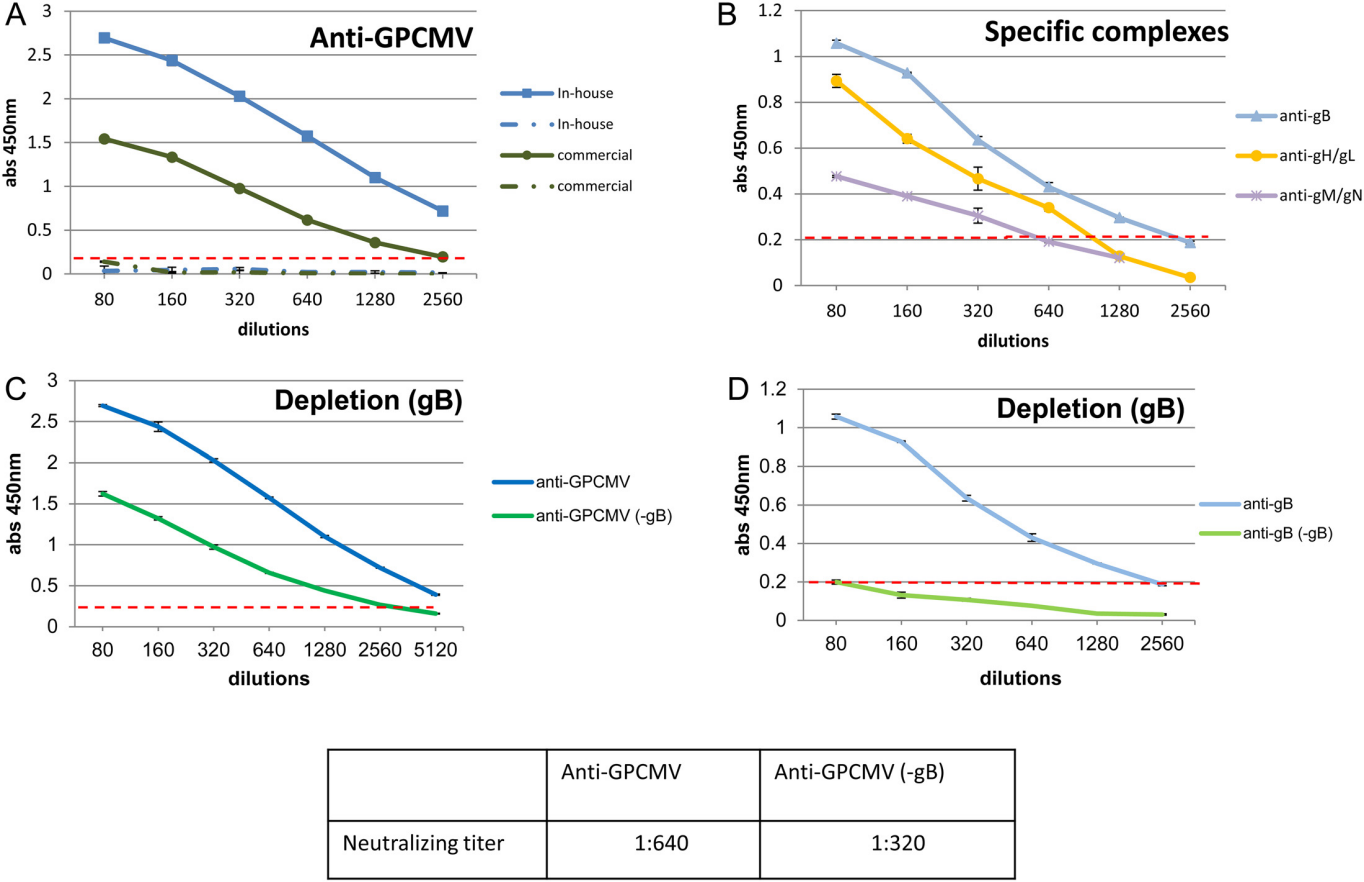
Antibody immune response of convalescent guinea pigs to GPCMV and GPCMV glycoprotein complexes determined by ELISAs. Pooled sera from GPCMV infected convalescent animals was used to evaluate immune response to GPCMV (A) or to specific glycoprotein complexes (B). (A) Anti-GPCMV ELISA. The immune response to GPCMV antigens was analyzed by an in-house ELISA (blue line square) compared to commercially available GPCMV ELISA kit from Bioexpress (green line circle). GPCMV sera negative for both assays are shown in blue and green dotted lines. (B) Immune response of convalescent pooled guinea pig serum to individual glycoprotein complexes. Anti-gB, light blue line (triangle); Anti-gH/gL, orange line (closed circle); Anti-gM/gN, purple line (star). Convalescent sera depleted of anti-gB antibody (-gB) (as described in materials and methods) was retested by ELISA to (C) anti-GPCMV ELISA (solid green line) to demonstrate retention of antibody response to other viral antigens, and (D) anti-gB specific ELISA (solid light green line) to demonstrate gB depletion compared to the original undepleted sera. All sera were diluted from 1:80 to 1:2560 in doubling dilutions. Control sera from animals negative for GPCMV were used for base line in all assays. ELISAs performed as described in materials and methods. Base level for background indicated by horizontal dotted red line.
