# Supplementary figures and images for: Viral Glycoprotein Complex Formation, Essential Function and Immunogenicity in the Guinea Pig Model for Cytomegalovirus
Source: PLoS One. 2015 Aug 12;10(8):e0135567. doi: 10.1371/journal.pone.0135567 (PMC4534421; doi:10.1371/journal.pone.0135567)

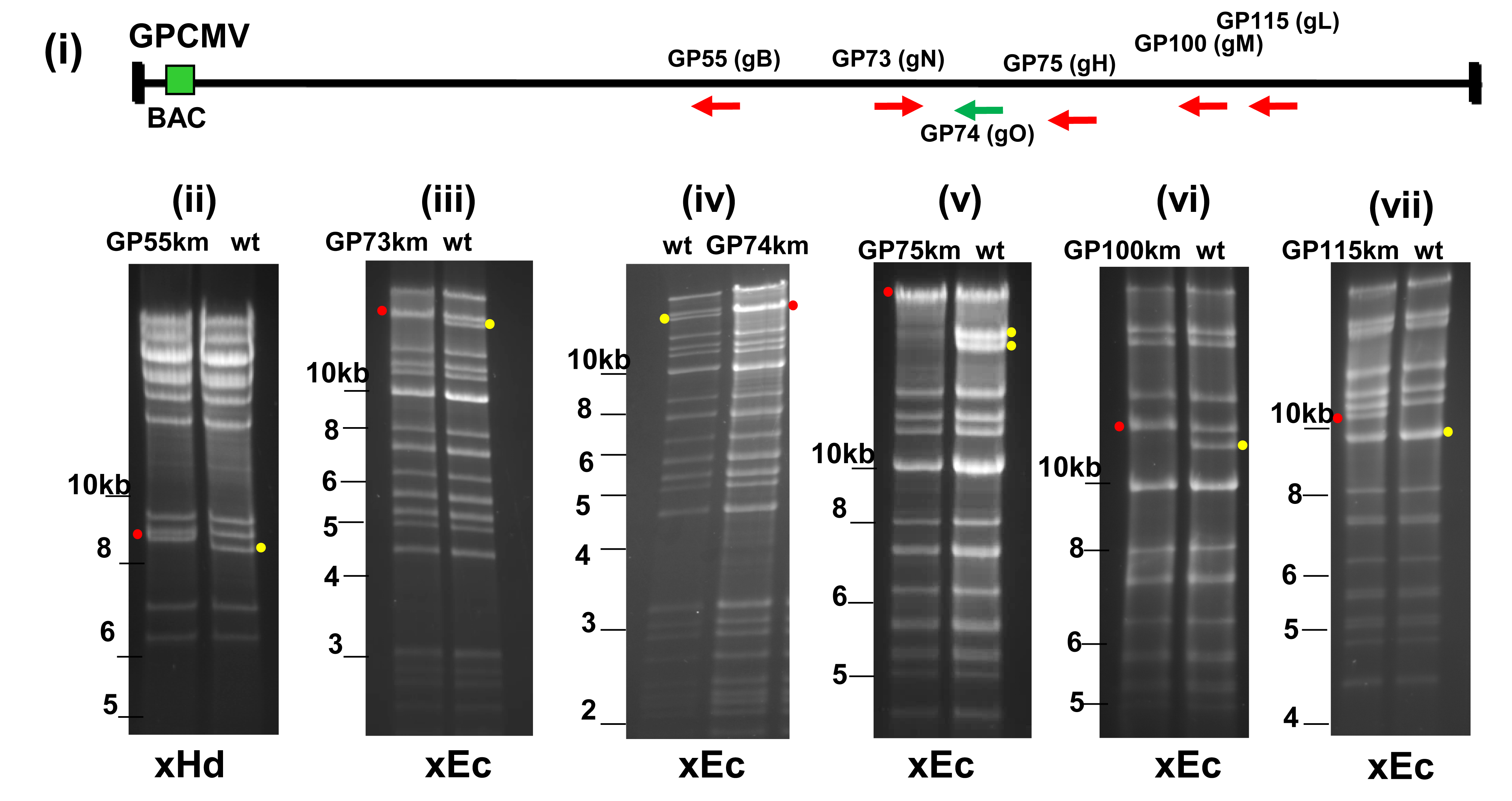

Supplement: S1 Fig — Wild type GPCMV BAC was mutated as described in materials and methods to individually knockout each glycoprotein gene in separate GPCMV BAC clones. At least two independent mutants were analyzed per gene knockout but only one mutant is shown in the Fig (identical results were obtained for the second mutant, data not shown). Both EcoR I (Ec) and Hind III (Hd) restriction profile analysis were performed for each mutant GPCMV BAC but only one profile is shown for each mutant to reduce repetition. Specific band shift are indicated as original wild type band (yellow) and modified mutant band (red). (i) Map of the GPCMV genome with individual glycoprotein genes indicated: GP55 (gB); GP73 (gN); GP74 (gO); GP75 (gH); GP100 (gM); GP115 (gL). Red indicates the gene is essential and green that the gene is semi-essential/non-essential for viable virus (see results in Fig 11). Individual GPCMV gene mutant and wild type BAC comparative restriction enzyme digests (ii)—(vii). Specific modification made to each gene in the process of inserting a kanamycin gene marker is shown in S2 Fig and PCR analysis of the modified locus shown in S3 Fig. (TIF) [file pone.0135567.s001.tif]

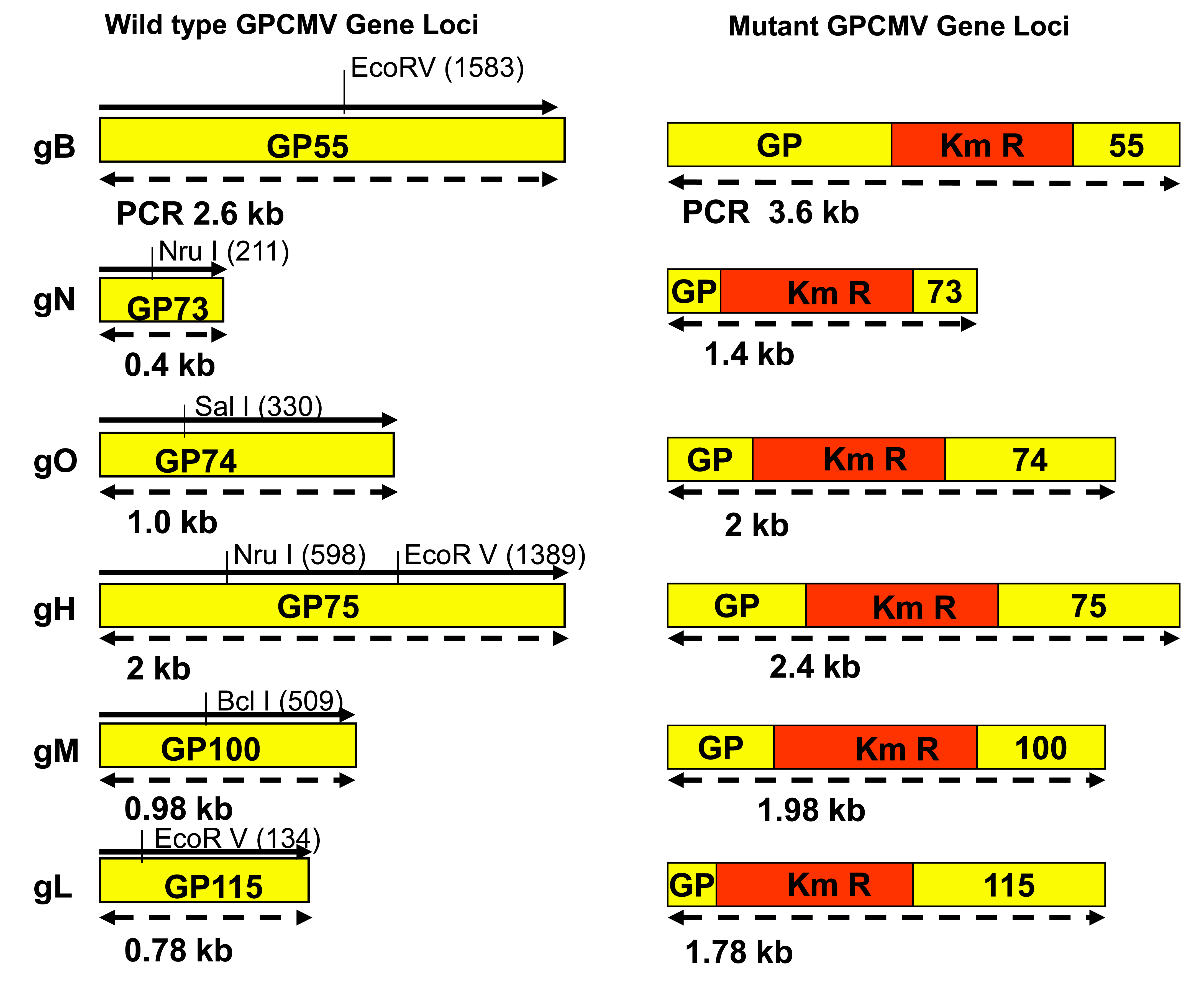

Supplement: S2 Fig — A kanamycin cassette was PCR amplified with modified restriction sites and cloned into individual glycoprotein knockout shuttle vectors. In the case of gN (GP73), gO (GP74), gH (GP75), gM (GP100) and gL (GP115) the entire ORF was cloned and modified by kanamycin cassette insertion using indicated restriction sites. The GP75 ORF was modified by a collapse between two sites (Nru I and EcoR V). For the gB (GP55) the homolog AD-1 domain was PCR cloned as a shuttle vector with Km inserted into a unique EcoR V site as described in materials and methods to disrupt the ORF. The sizes of the original genes by PCR analysis are indicated and the sizes of the modified genes after kanamycin cassette insertion are also indicated (sizes verified by PCR in S4 Fig). (TIF) [file pone.0135567.s002.tif]

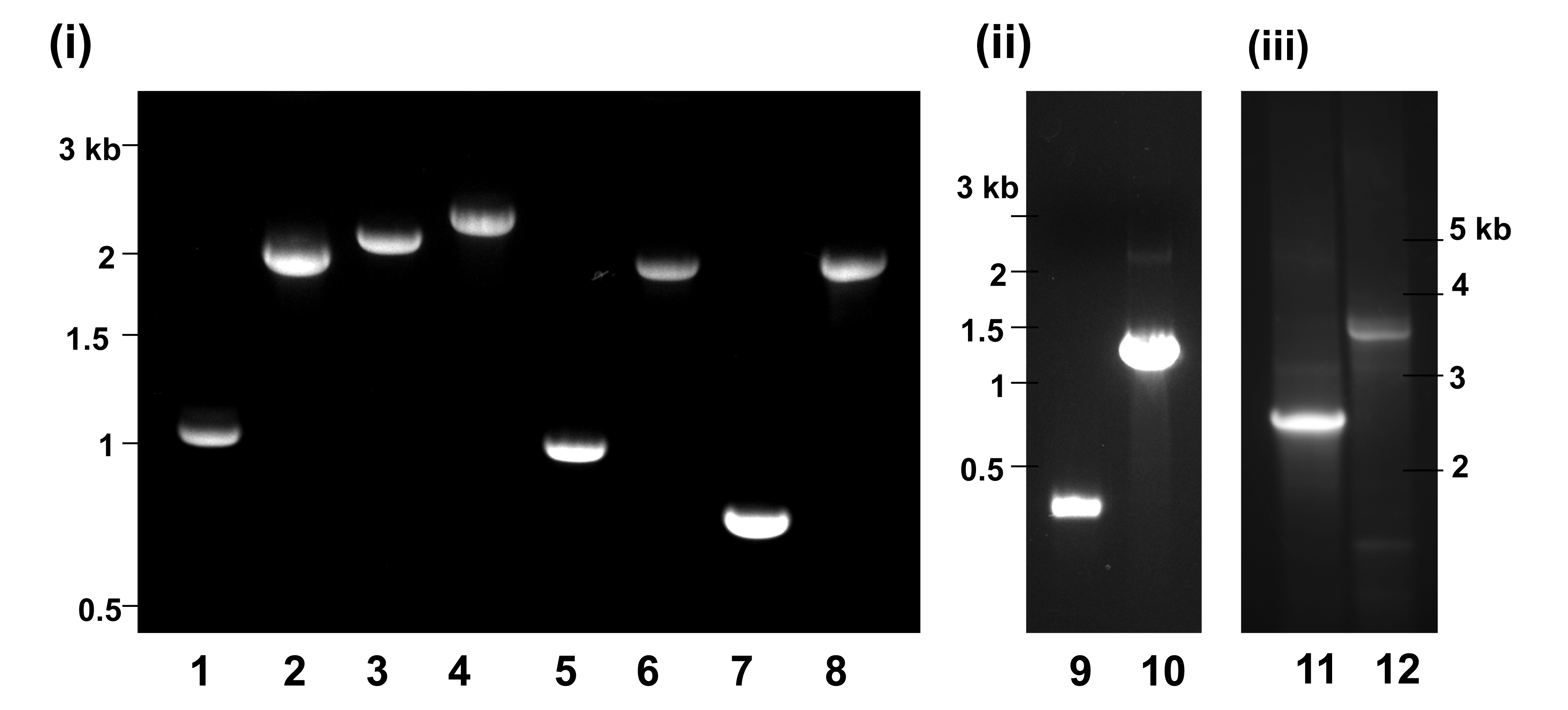

Supplement: S3 Fig — Common primers were used to amplify the genes of wild type and mutant GPCMV. PCR primers as described in materials and methods and S1 Table were used to verify that the individual glycoprotein genes had been correctly modified. PCR products of mutant and wild type genes were compared by agarose gel electrophoresis to verify specific modifications. Gels: (i) GP74, GP75, GP100, GP115. (ii) GP73. (iii) GP55. GPCMV BAC mutant and wild type GPCMV analysis via PCR. Sample lanes: (1) GP74 wt; (2) GP74 mutant; (3) GP75 wt; (4) GP75 mutant; (5) GP100 wt; (6) GP100 mutant; (7) GP115 wt; (8) GP115 mutant (9) GP73 wt; (10) GP73 mutant; (11) GP55 wt; (12) GP55 mutant. (TIF) [file pone.0135567.s003.tif]

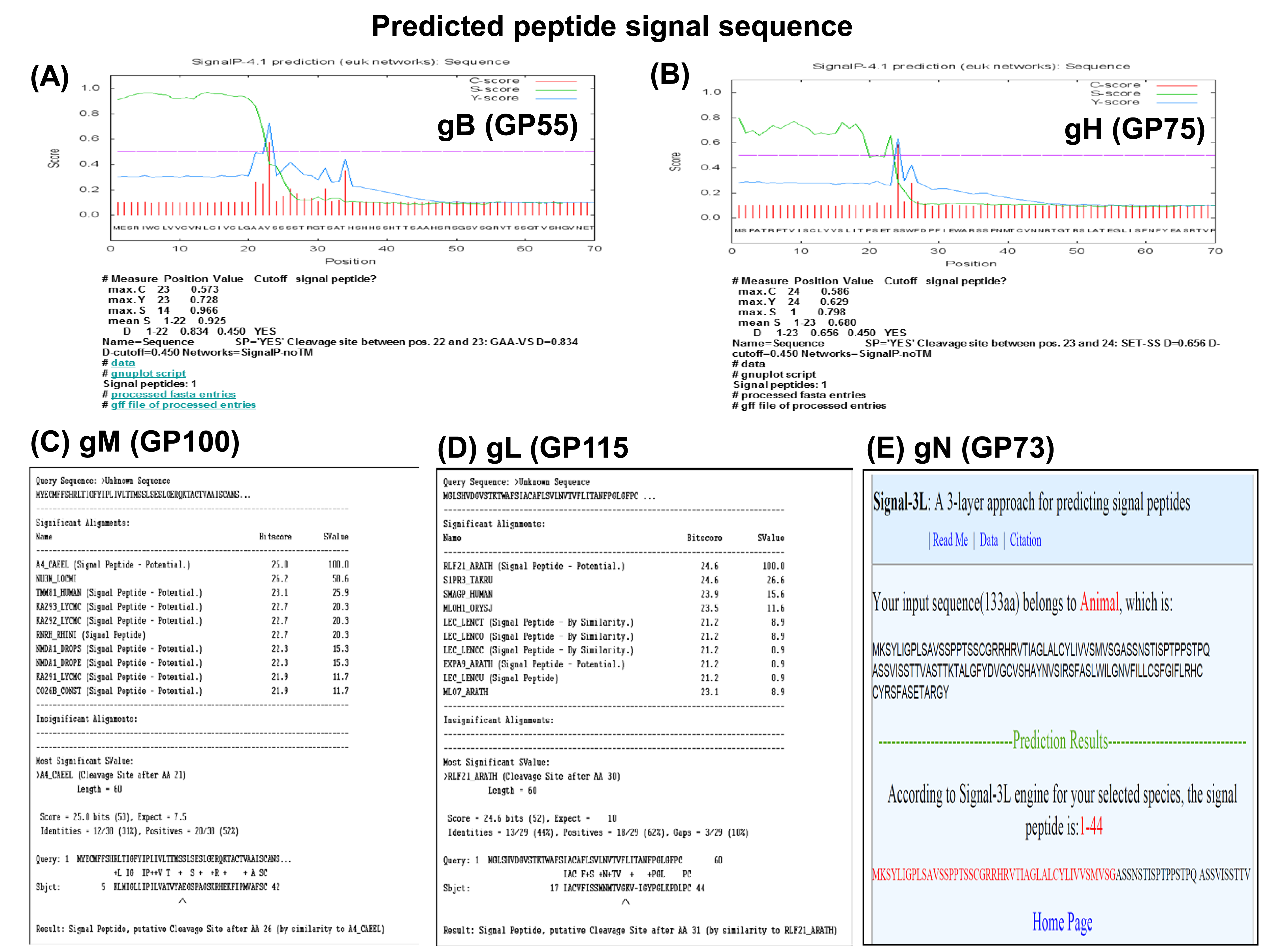

Supplement: S4 Fig — Various web based programs were used to predict the presence of a signal peptide sequence associated with individual proteins. (A) gB and (B) gH leader sequences determined by http://www.cbs.dtu.dk/services/SignalP/ [48]. (C) gM and (D) gL leader sequences determined by http://sigpep.services.came.sbg.ac.at/signalblast.html. (E) gN leader sequence determined by http://www.csbio.sjtu.edu.cn/bioinf/Signal-3L/ [49]. Data shown is the end result analysis from each program. (TIF) [file pone.0135567.s004.tif]

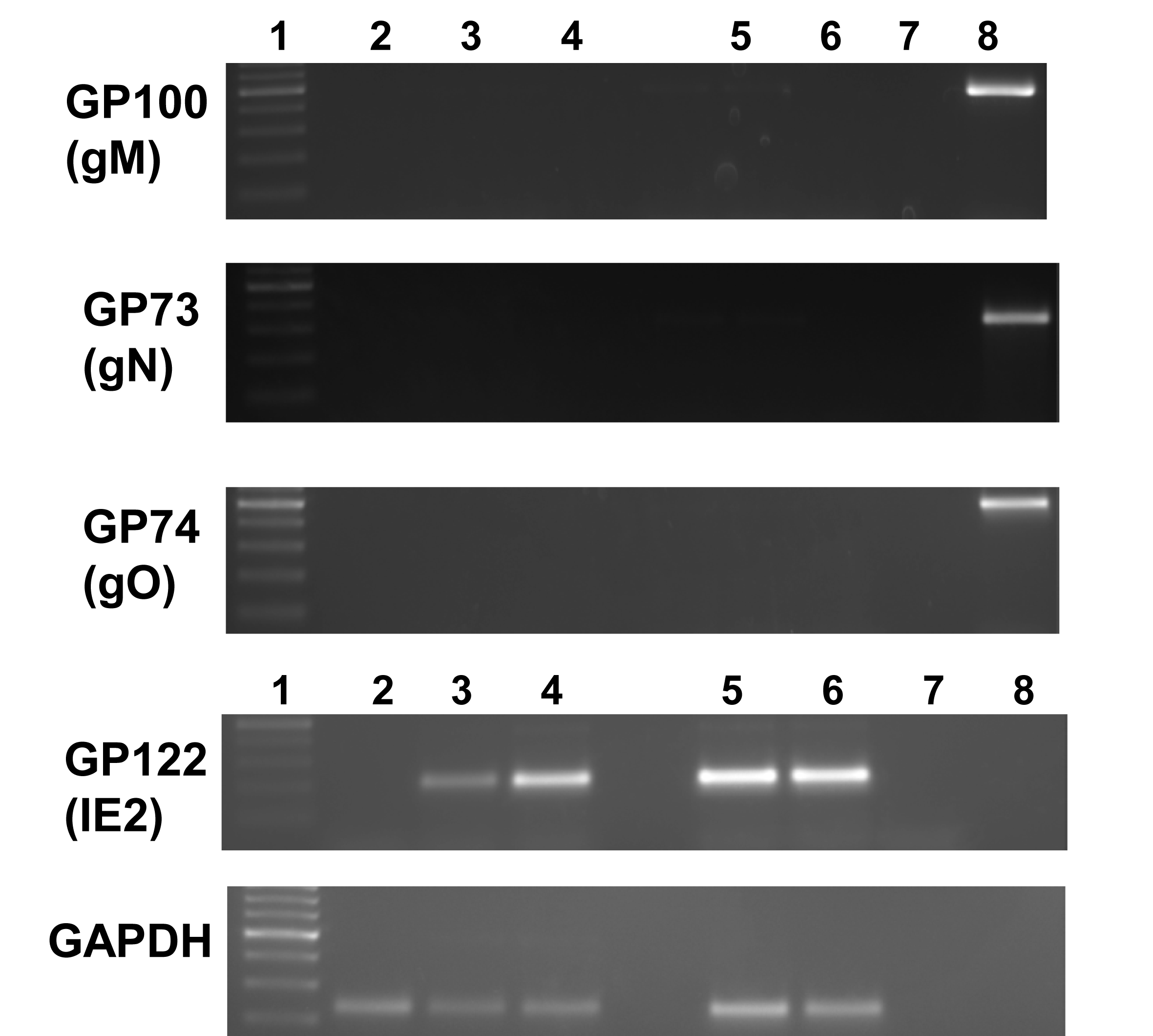

Supplement: S5 Fig — RT-PCR assays were carried out with GPCMV strain 22122 infected GPL cells in 6 well dish (moi = 1 pfu/cell) at different time points (6, 24 and 48 hr post infection) in the presence or absence of specific chemical inhibitors. Cycloheximide (CHX, 100 μg/ml) was used to prevent transcription of all but the IE transcripts and phosphonoacetic acid (PAA, 200 μg/ml) was used to prevent late transcripts as described in materials and methods. RT-PCR was carried out as described in materials and methods. Lanes: 1, bp ladder (Invitrogen); 2, mock infected; 3, 6 hour CHX treated; 4, 24 hour CHX treated; 5, 24 hour PAA treated; 6, 48 hour PAA treated; 7, no template control; 8, infected cell lysate no reverse transcriptase stage; 9, untreated (no inhibitor) GPCMV infected cell lysate. GP122 (IE2) RT-PCR is a positive control for GPCMV at specific assay time points treated with inhibitors. GAPDH is a positive cellular RNA control for all time point samples. (TIF) [file pone.0135567.s005.tif]

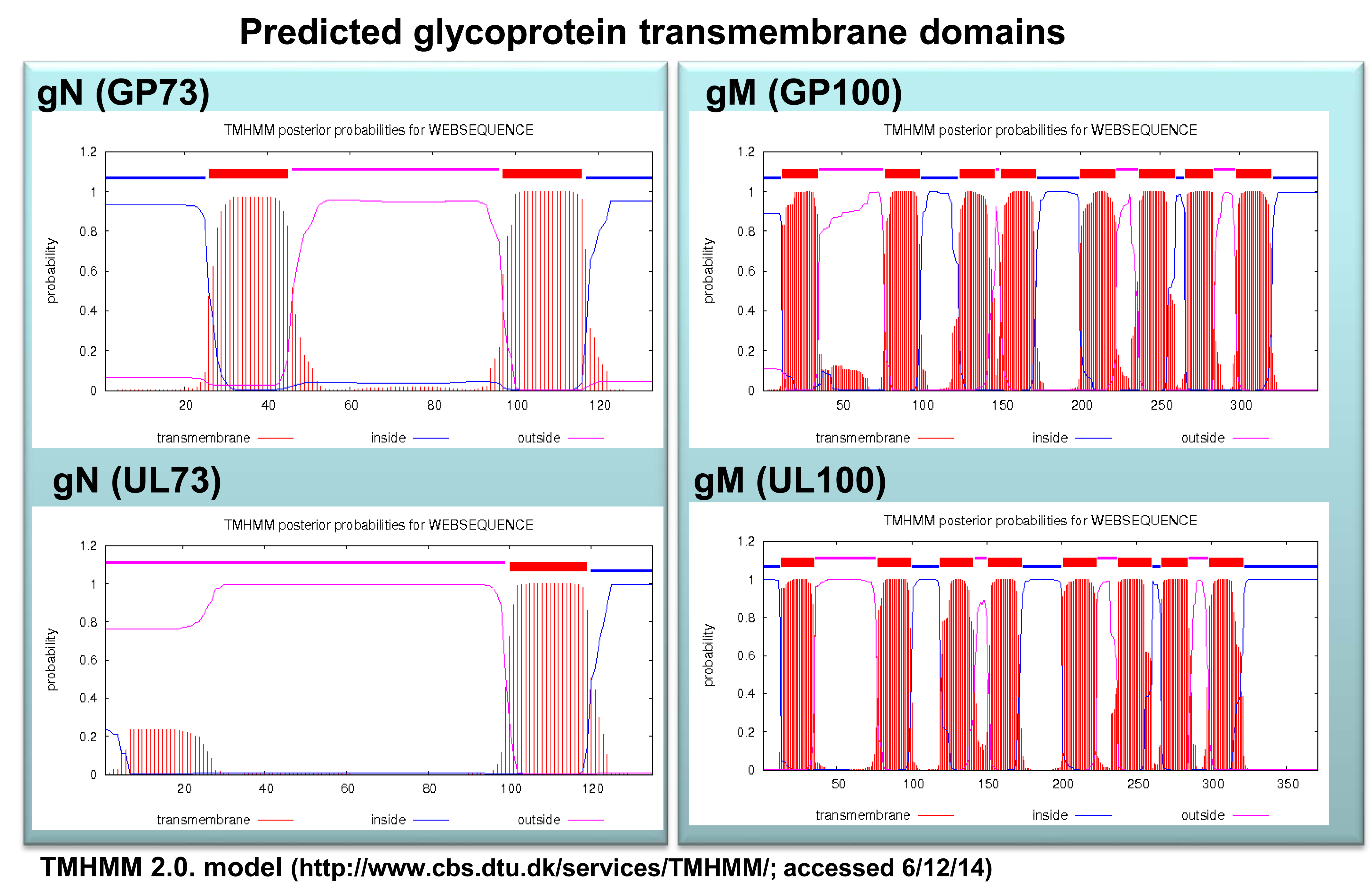

Supplement: S6 Fig — The predicted amino acid sequences for HCMV and GPCMV gM and gN proteins were analyzed for potential transmembrane domains by the web based program TMHMM Server v. 2.0 Prediction of transmembrane helices in proteins (http://www.cbs.dtu.dk/services/TMHMM/). Potential transmembrane helices indicated in red in alignment with the predicted protein sequence (N to C terminal). (TIF) [file pone.0135567.s006.tif]

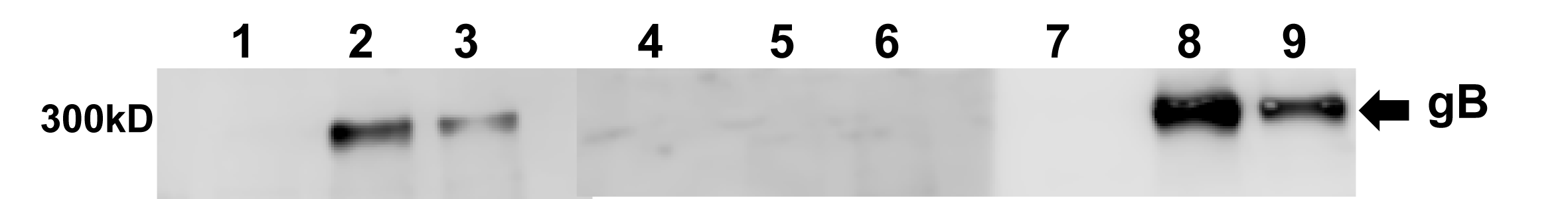

Supplement: S7 Fig — Anti-GPCMV sera depleted for anti-gB antibodies by preabsorption against Ad-gB transduced HEK 293 cells was verified for depletion by Western blot analysis as described in Materials and Methods. Lanes 1, 4, 7 mock infected GPL cells; Lanes 2, 5, 8 Ad-gB transduced GPL cell lysates (moi = 20 TDU/cell); Lanes 3, 6, 9 late stage GPCMV infected GPL cell lysates (moi = 1 pfu/cell). GPCMV convalescent sera (1:500) used for lanes 1–3, anti-gB depleted GPCMV sera (1:100) used for lanes 4–6. GPCMV gB monoclonal antibody (29–29) used for lanes 7–9 (1:500). Black arrow shows gB. (TIF) [file pone.0135567.s007.tif]
