# Supplementary material for: Viral Glycoprotein Complex Formation, Essential Function and Immunogenicity in the Guinea Pig Model for Cytomegalovirus
Source: PLoS One. 2015 Aug 12;10(8):e0135567. doi: 10.1371/journal.pone.0135567 (PMC4534421; doi:10.1371/journal.pone.0135567)
Supplement: S1 Table — (DOC) [file pone.0135567.s008.doc]

**S1 Table. Oligonucleotides used for PCR and RT-PCR analysis of GPCMV glycoprotein genes.**

| **Oligo (gene)** | **Sequence** |
| --- | --- |
| GP55FD (GP55) | 5’ GCAGTTGGCGAGGGACGTGGACATC |
| GP55R (GP55) | 5’ GCACGTAGCATCCGCACGGCGT |
| FGP73 (GP73) | 5’ CTATCGGCTGTGTCATCGCCG |
| RGP73 (GP73) | 5’ CTCTGGCGGTCTCGCTGGCAAACG |
| FGP74 (GP74) | 5’ GTTGATAGCATCTGTCTATCCGACTTCG |
| RGP74 (GP74) | 5’ CAGCGAGACCGCCAGAGGTTACTAAAGG |
| FGP75 (GP75) | 5’ CTGATCACGCCGTCGGAGACTTGGTC |
| RGP75 (GP75) | 5’ GTATAGCAGCAATACGCCGAGCGAGCAG |
| GP100F (GP100) | 5’ CTCTCTCACGTGGACGGGGTGAGCAC |
| GP100R (GP100) | 5’ GTCTATGTCCAACTTGGACGAGAG |
| FGPgL (GP115) | 5’ ATGTATGAATGTATGTTTTTTTCGCATCGT |
| RGPgL (GP115) | 5’ TCAGCTCAGATCCCTGTTTAAGTAATTGTG |
| KmF | 5’ CGATTTATTCAACAAAGCCACG |
| KmR | 5’ GCCAGTGTTACAACCAATTAACC |
| Fgp55Bgl (GP55) | 5’AGATCTATGCGACCCGTACGCGGTATCGCGAGATC |
| Rgp55Bgl (GP55) | 5’AGATCTTTACGGCGGTATAGGATTGATGACCCGTTCAACC |
| Rgp55fullBgl (GP55) | 5’AGATCTTTAAACATGCACGTCGTCTTCCGTGTTAATTTTTGATAACCTCCTC |
| FgBEc (GP55) | 5’ATCATAGAATTCGTATGACATGGATGGAAATGCGACGTATCATG |
| RgBPst (GP55) | 5’GATACACTGCAGACGAGGACGACACTCGAGTGTCCGCATCCACG |
| FgHBm (GP75) | 5’GGATCCATGTCACCCGCGACGCGGTTTACC |
| RgHBmNostop (GP75) | 5’GGATCCAGAATCATATGTAAGAGGCGGTATAGACCGTATAG |
| FgLHd (GP115) | 5’AAGCTTATGTATGAATGTATGTTTTTTTCGCATCGT |
| RgLHdNostop (GP115) | 5’AAGCTTGCTCAGATCCCTGTTTAAGTAATTGTG |
| FGP74EcV (GP74) | 5’GATATCATGGTGTTATTTTTGTACCCGTTGATAG |
| RGP74XhoNostop (GP74) | 5’CTCGAGAGGCACTGCTGTTACCGGTCGTTTGCCAG |
| gMTagF (GP100) | 5’TAGCAGAATTCATGGGGCTCTCTCACGTGGACGGGGTGAGCAC |
| gMTagR (GP100) | 5’TGCATCGGATCCGCCTCGTCTATGTCCAACTTGGACGAGAG |
| gNTagF (GP73) | 5’TATGCAGAATCATGAAGAGTTATCTTATAGGGCCCCTATCGGCTGTG |
| gNTagR (GP73) | 5’TGCTCAGGATCCGCGTAACCTCTGGCGGTCTCGCTGCAAACG |
| FGP73BmShort (GP73) | 5’ATAGATGGATCCATGGTGTCGGGGGCGAGTTCCAATAGC |
| FGP73BmFull (GP73) | 5’ATAGATGGATCCATGAAGAGTTATCTTATAGGGCCCCTATC |
| RGP73nostopHd (GP73) | 5’ATAGATAAGCTTGTAACCTCTGGCGGTCTCGCTGGCAAAC |
| GP73RTF (GP73) | 5’GTCATGTGGTCGACGACAC |
| GP73RTR (GP73) | 5’GTAACAGCAGTGCCTTAG |
| GP74RTF (GP74) | 5’CGAGACGCAACCGCGACGGAC |
| GP74RTF (GP74) | 5’GCATCTGTCTATCCGACTTCG |
| GP100RTF (GP100) | 5’GACTGATATTGCGTGATCAGG |
| GP100RTR (GP100) | 5’GCGTCCTGAACGTCACGGTG |
| GAPDHRTF (GAPDH) | 5’GGGCAAGGTCATCCCAGAG |
| GAPDHRTR (GAPDH) | 5’TGGAAGAATGGCTGTCACTGTT |
| GP122RTF (GP122) | 5’ACTGCTGGGTCTCACGC |
| GP122RTR (GP122) | 5’CATTTGATGCTATGGACAGGGACT |
